# FOXM1 is critical for the fitness recovery of chromosomally unstable cells

**DOI:** 10.1038/s41419-023-05946-2

**Published:** 2023-07-14

**Authors:** Fan Pan, Sara Chocarro, Maria Ramos, Yuanyuan Chen, Alicia Alonso de la Vega, Kalman Somogyi, Rocio Sotillo

**Affiliations:** 1 Division of Molecular Thoracic Oncology, German Cancer Research Center (DKFZ), Im Neuenheimer Feld 280, 69120 Heidelberg, Germany; 2 Ruprecht Karl University of Heidelberg, Heidelberg, Germany; 3 German Center for Lung Research (DZL), Translational Lung Research Center Heidelberg (TRLC), Heidelberg, Germany; 4 German Consortium for Translational Cancer Research (DKTK), 69120 Heidelberg, Germany

**Keywords:** Cell biology, Cancer

## Abstract

Tumor progression and evolution are frequently associated with chromosomal instability (CIN). Tumor cells often express high levels of the mitotic checkpoint protein MAD2, leading to mitotic arrest and cell death. However, some tumor cells are capable of exiting mitosis and consequently increasing CIN. How cells escape the mitotic arrest induced by MAD2 and proliferate with CIN is not well understood. Here, we explored loss-of-function screens and drug sensitivity tests associated with MAD2 levels in aneuploid cells and identified that aneuploid cells with high MAD2 levels are more sensitive to FOXM1 depletion. Inhibition of FOXM1 promotes MAD2-mediated mitotic arrest and exacerbates CIN. Conversely, elevating FOXM1 expression in MAD2-overexpressing human cell lines reverts prolonged mitosis and rescues mitotic errors, cell death and proliferative disadvantages. Mechanistically, we found that FOXM1 facilitates mitotic exit by inhibiting the spindle assembly checkpoint (SAC) and the expression of Cyclin B. Notably, we observed that FOXM1 is upregulated upon aneuploid induction in cells with dysfunctional SAC and error-prone mitosis, and these cells are sensitive to FOXM1 knockdown, indicating a novel vulnerability of aneuploid cells.

## Introduction

Chromosomal instability (CIN), defined as the continuous loss or gain of chromosomes is the result of increased levels of mitotic errors and aneuploidy [1]. Although CIN promotes tumor evolution, drug resistance and tumor heterogeneity, excessive CIN leads to cell death [2–4]. The mechanisms conferring cancer cells tolerance to high levels of CIN are not fully understood [5]. The identification of specific targets against these bypass mechanisms could provide therapeutic strategies against CIN tumors [6, 7].

The spindle-assembly checkpoint (SAC) stalls the onset of anaphase until all kinetochores are properly bounded to microtubules during metaphase, preventing chromosome errors and CIN [8, 9]. Overexpression of SAC proteins is common among human cancers and defective SAC functioning facilitates ongoing CIN [10, 11]. Among these SAC proteins, MAD2 has been found to be upregulated in different types of cancer and its overexpression, in transgenic mouse models, induces mitotic arrest and increases the number of mitotic errors [12]. In addition, Mad2 overexpression in Kras-driven mouse breast tumors leads to increased somatic copy number alterations compared with Kras tumors [13]. However, how tumor cells overcome Mad2-induced mitotic arrest and tolerate Mad2-induced CIN is still unclear. Understanding the molecular mechanism behind could reveal novel therapeutic strategies for unstable MAD2-overexpressing cancers.

Microtubule (MT) poisons block mitosis by interfering with microtubule dynamics and therefore activating the SAC and have been widely applied in the treatment of solid cancers [14, 15]. Intriguingly, clinical trials indicate that the cytotoxic effect of microtubule-targeting drugs might not only rely on the induced mitotic arrest but also in the excessive CIN generation [16].

One key mediator of anti-mitotic therapeutic response is the Forkhead Box M1 (FOXM1) transcription factor, which is involved in mitotic progression, spindle assembly and chromosome segregation [17–19]. FOXM1 preserves mitotic spindle formation and prevents mitotic catastrophe induced by the MT poison paclitaxel [20] while repression of FOXM1 increases paclitaxel-induced mitotic cell death through modulation of the apoptotic pathway [21]. FOXM1 also improved age-associated mitotic defects in elderly human dermal fibroblasts, leading to decreased aneuploid levels [22].

Here, we identify FOXM1 to be essential for the survival of tumor cells with MAD2 overexpression (OE) in mouse and human cell lines. FOXM1 overexpression facilitates mitotic exit and maintains chromosome segregation fidelity of MAD2-overexpressing and nocodazole-treated cells by disrupting SAC signaling. Analysis of human tumors showed that high FOXM1 expression and increased aneuploidy were associated with poor prognosis, and cells with tetraploidization were more sensitive to depletion of FOXM1. Our results revealed that the upregulation of FOXM1 is a mechanism that allows cells to bypass mitotic arrest and tolerate CIN in MAD2-overexpressing cells.

## Materials and methods

### Human cancer cell lines data

Aneuploidy score (AS) and gene expression data sets were obtained from DepMap portal (https://depmap.org/portal/). Aneuploid and near euploid cell lines were split into two groups: the top and bottom quartiles of AS. Aneuploid cell lines were further separated as top and bottom sextile of MAD2 levels, according to the MAD2 expression levels to gain further insight into specific genes that are essential in the MAD2_high cell lines. Genetic dependency data sets were CRISPR (DepMap 22Q2 Public + Score, Chronos) and RNAi (Achilles + DRIVE + Marcotte, DEMETER2). Essential genes in high or low MAD2 aneuploid compared to near euploidy were determined by using R package Limma. The different dependency of each gene between groups was evaluated and considered as significant gene when the fold change of mean dependency was above 1 and the *P*-value was <0.01. *P*-values were derived from Wilcoxon t-test. To compare AS or gene expression among groups, we applied Prism9 to generate scatterplots. Drug sensitivity data sets were obtained from drug sensitivity (AUC) [23] and Drug Repurposing Secondary Screen 19Q4 [23]. The sensitivity of drug screens was compared among groups. *P-*values were determined by one-way ANOVA. EC50 was compared between euploidy and aneuploidy with high or low MAD2, respectively. *P*-values were determined by Wilcoxon t-test.

### GEO and TCGA human cancer data analysis

Gene expression of human breast cancer data sets was obtained from Gene Expression Omnibus (GEO) datasets (http://www.ncbi.nlm.nih.gov/geo). Two groups of samples were determined as the top and bottom sextile of MAD2 levels.

Copy number alterations and gene expression (FPKM-UQ) of patient samples from TCGA were obtained from UCSC Xena functional genomics explorer. Data of mean absolute change in copy number segment were filtered to remove the bottom quartile across all segment lengths to better obtain ploidy changes. Top and bottom quartiles of mean absolute change were considered as aneuploid and euploid cancers. Comparison of genes between groups was done by Limma package. Genes with >2.5-fold change of expression and <0.01 of *P-*value were considered as significant. *P*-values were determined by Wilcoxon t-test.

### Mouse models and Xenograft experiment

*KH2-HA-Mad2*, *TetO-Mad2*, *TetO-Kras*^*G12D*^, *MMTV-rtTA* and *H2B-GFP* mice were generated as described previously [13]. All animals were in FVB background and housed in specific pathogen-free conditions. Breeding and experiments were performed at DKFZ animal facilities under permit numbers G231/15 and G18/21 from Regierungspräsidium of Karlsruhe, Germany. To induce transgenes, 8-week-old female mice were administrated with doxycycline via impregnated food pellets (625 mg/kg; Harlan-Teklad). Tumor growth was regularly examined and tumors were collected when they reached 1.5 cm^3^. Xenografts of Balbc nude mice: 250,000 MCF-7 cells in 0.1 mL serum-free medium were injected subcutaneously in 6-week-old female mice. Nude mice were administrated with doxycycline food 3 days before cell injection. Tumors were measured every two days and harvested when size reached 1.5 cm^3^. Tumor size was calculated with formula length (*L*) × width (*W*)^2^/2. No statistical method was used to estimate the sample size of the mouse models or xenograft mice. The sample size was based on the previous experimental observations [13]. No data were excluded from the animal experiment. The researchers were blind to the allocation of the animal groups during the experiment.

### RNA-seq analysis of mouse tumor samples

RNA-seq data was obtained from the European Nucleotide Archive (ENA; http://www.ebi.ac.uk/ena) under number PRJEB13611 [24]. The data matrix was processed with DEseq2 VST normalization. Comparison of gene expression between K and KM tumors was analyzed via Lima package. DEGs were identified if log2 fold changes >1.0 and *P* < 0.05. Metascape was used to perform pathway analysis. Significant pathways were identified if *P* < 0.05 and FDR < 0.5.

### Cell culture and in vitro experiments

Mouse breast tumor cells were harvested from *KH2-HA-Mad2*, *TetO-Kras*^*G12D*^, *MMTV-rtTA*; *TetO-Mad2*, *TetO-Kras*^*G12D*^, *MMTV-rtTA* and from *TetO-Kras*^*G12D*^, *MMTV-rtTA* and cultured as previously published [13]. MEFs were prepared from *KH2-HA-Mad2/Rosa26-rtTA* and maintained using procedures described in [12]. MDA-MB-231 (RRID: CVCL_0062), MCF7 (RRID:CVCL_0031) and CAL51 (RRID: CVCL_1110) cells were cultured in DMEM (Life Technologies, 419650394) with 10% fetal bovine serum, Tetracycline free (VWR, S181T-500) and 1% penicillin-streptomycin (Life Technologies, 15140122). MCF10A (RRID: CVCL_0598) cells were maintained as described in [25]. All cell lines have been authenticated using STR profiling. All experiments were performed with mycoplasma-free cells. To generate MAD2 and FOXM1 inducible cell lines, cells were infected with rtTA or rtTA-GFP-expressing retrovirus. Selection was performed with puromycin (1 μg/ml) or FAC sorting, then infected with inducible Tet-ON lentiviruses carrying human MAD2 and FOXM1 cDNA (MAD2 from Addgene #136347; FOXM1b from Addgene #68811; FOXM1c from Addgene #68810) and selected with hygromycin (300 μg/ml) or puromycin (1 μg/ml). Doxycycline concentration in all cultured experiments was 1 μg/ml. To inhibit FOXM1, 200 μM siRNA and lipofectamine 2000 transfection reagent (Invitrogen) were prepared in Opti-MEM reduced-serum media (Gibco). Cells were treated with siRNA for the indicated number of days. The siRNA used were *FOXM1* siRNA (human) (sc-37615), *Foxm1* siRNA (mouse) (sc-44877), Control siRNA (sc-37007).

Cell cycle profile was performed by counterstaining DNA with propidium iodide (Life Technologies, BMS500PI) and analyzed by a FACS-Canto flow cytometry device (BD Biosciences). TUNEL staining was done following manufacture instructions (Roche, 12156792910). Cell proliferation was determined with the Promega, G3580 kit following manufacture instructions. For immunostaining, cells were first harvested in a dilute suspension and deposited onto a slide with a cytospin centrifuge, according to protocols (Thermo, TH-CYTO4). Then cells were fixed with 4% paraformaldehyde (VWR, J61899.AP). Blocking was done using 10% donkey or goat serum (Jackson Immuno) in PBS with 0.15% triton X. Primary antibodies were : FOXM1 (1:500, Abcam, ab207298), MAD2 (1: 500, BD biosciences, 610678), HA (1:1000, Covance, MMS-101R), FLAG (1:500, Sigma, F7425), pH3 (1:1000, Cell signaling, 9701 S), Aurora B (1:500, BD biosciences, 611082). Secondary antibodies were Alexa fluorophore-labeled donkey/goat IgG (1:500, Invitrogen). Images were taken in a Leica SP5 confocal microscope and Tissuegnostic TissueFAX system and analyzed using ImageJ. Time-lapse imaging was performed for at least 12 h using a time-lapse microscope 2 μm optical sectioning across 20 μm stack, every 5 min.

Concentrations of nocodazole (Sigma, 487928) were 200 ng/ml, as indicated in figure legends. Tetraploid MCF7 and CAL51 were generated by cytokinesis inhibition using 0.75 μM dihydrocytochalasin B (DCB, inhibitor of actin polymerization, Sigma-Aldrich D1641) for 18 h overnight. Afterwards, cells were washed 3 times with PBS and cultured in DMEM supplemented with 10% FBS and 1% penicillin-streptomycin for an additional 20 h.

### Three-dimensional organotypic assays

Mammary epithelial cells were harvested from 8-week-old female mice. 3D cell culture and staining were performed according to [13]. Primary antibodies were HA (1:1000, Covance, MMS-101R), FOXM1 (1:500, HUABIO, ER 1706-62), Images were taken with a Leica TCS SP5 confocal microscope and analyzed using ImageJ.

### Quantitative PCR

Frozen tissue from mice was ground with mortar and pestle on dry ice and RNA was purified using RNeasy Mini Kit (Qiagen). To synthesize cDNA, we used QuantiTect Reverse Transcription Kit (Qiagen). For real-time quantification, 8 ng cDNA was used as a starting material along with SYBR Green PCR Master Mix (2x) (Applied Biosystems) in a LightCycler II ^®^ 480 (Roche). PCR program : 95 °C for 5', 45x [95 °C for 10'', 60 °C for 15'', 72 °C for 15''], [95 °C for 5', 65 °C for 1’]; Formulas for calculation of gene expression: ΔCt = Ct (gene of interest) – Ct (reference gene); ΔΔCt = ΔCt – ΔCt (reference sample); Primers were Actin F: GCTTCTTTGCAGCTCCTTCGT and Actin R: ACCAGCCGCAGCGATATCG; FOXM1 F: GCGTTAAGCAGGAACTGGAA and FOXM1 R: TCAGACACAGAGTCCTGCCA.

### Western blot

40 μg of cell lysates obtained from ground-frozen tissue or cells were used for assessing protein expression. Primary antibodies used for detection were FOXM1 (1:1000, Abcam, ab207298), MAD2 (1:1000, BD biosciences, 610678), HA (1:1000, Covance, MMS-101R), Cyclin B (1:200, Santa Cruz, SC-245), Cleaved-Caspase 3 (1:1000, Cell signaling, 9661), p-Histone H2AX (1:250, Santa Cruz, SC-517348), CDC20 (1:250, Santa Cruz, SC-13162), Actin (1:3000, Sigma, A2066). Protein band quantification was carried out using ImageJ.

### Statistical analysis

Prism9 was used for statistical analysis. Statistical analyses between two groups were carried out using unpaired *t*-test, between more than two groups were performed with one-way analysis of variance, followed by Tukey’s multiple comparisons test or two-way analysis of variance, followed by Sidak’s multiple comparisons test. *P-*values were indicated in figure legends. *P*-value < 0.05 was considered as significant. Scatterplots were shown as mean and SEM. Points and connecting lines were shown as mean and error with SEM. Cell number and animal number were presented as *n*.

## Results

### High levels of MAD2 confer aneuploid cells sensitivity to FOXM1 inhibition

To understand how the genetic landscape in human tumors is altered by MAD2 expression, we performed independent analysis from data obtained from a human cancer cell line dataset (DepMap, *n* = 1389) and human breast tumors (GSE102484, *n* = 683; GSE54002, *n* = 417; GSE76275, *n* = 265). Cancer cell lines and GSE tumors were divided into sextiles according to MAD2 expression levels. Differential expression analysis between the top and bottom sextiles revealed 62 differentially expressed genes (62 DEG) that were frequently upregulated in all MAD2_high cancer cell lines and MAD2_high tumors (*P* < 0.01; log2 fold change > 1.5) (Supplementary Fig. 1A).

**Fig. 1:**
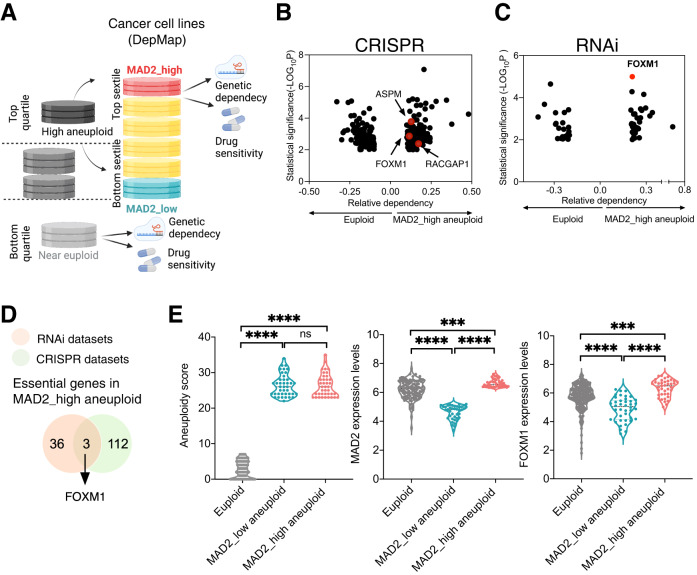
Identification of FOXM1 as a vulnerability of MAD2 high aneuploid cells. **A** Schematic of our comparison of genetic and chemical dependencies between MAD2 high and low cancer cell lines. Cell lines were assigned aneuploidy scores (AS), and the genetic and drug sensitivity landscapes were compared between the top and bottom AS quartiles. Cell lines in the top quartile of aneuploidy scores were divided into top and bottom sextiles according to their MAD2 expression (MAD2_high aneuploid vs near euploid cell lines and MAD2_low aneuploid vs near euploid cell lines). **B** Essential genes in MAD2_high aneuploid cells when compared to euploid cells, based on a CRISPR-Cas9 screen. The unique differential genetic dependencies in MAD2_high and euploid groups are shown in a volcano plot. The three genes present in both DEGs and CRISPR datasets are highlighted in red. **C** Essential genes for MAD2_high and euploid cell lines in RNAi datasets are shown as relative dependency. FOXM1 is highlighted in red. **D** The number of essential genes of MAD2_high aneuploid in RNAi and CRISPR datasets. **E** Comparison of aneuploidy scores and mRNA expression levels of *MAD2* and *FOXM1* between euploid and aneuploid cancer cell lines. ****P < 0.0001 and ***P = 0.0002; One-way ANOVA.

Next, to understand how cells with high levels of MAD2 are able to tolerate CIN, we investigated the vulnerabilities of aneuploid MAD2_high cell lines. Cancer cell lines from DepMap were divided into quartiles based on their aneuploidy score (AS) [26]. Aneuploid cell lines (Top AS quartiles) were further distributed into sextiles according to their MAD2 levels. MAD2_high (Top sextile) and MAD2_low (Bottom sextile) groups were considered for further analysis. The cell lines in the low AS quartile were considered the near euploid group. Based on this stratification, we performed a comparison of genetic dependency between MAD2_high aneuploid (aneuploid with high levels of MAD2) and euploid cell lines (Fig. 1A). Analysis of CRISPR-Cas9 datasets revealed that 115 genes were essential in MAD2_high aneuploid cells (Fig. 1B, Table 1) [26]. Three genes (*RACGAP1*, *ASPM*, *FOXM1*) were present in both DEGs and CRISPR datasets (Supplementary Fig. 1B). We then investigated RNAi (Achilles+DRIVE) datasets to look for genes whose depletion was more lethal to MAD2_high aneuploid cell lines than to euploid ones. We identified 39 differential dependencies of MAD2_high aneuploid cells (Fig. 1C, Table 1) and confirmed that *FOXM1* was a common dependency for MAD2_high aneuploid cells in both RNAi and CRISPR datasets (Fig. 1D). Further analysis showed that MAD2_high aneuploid cells exhibited increased *FOXM1* mRNA levels compared to euploid cells or MAD2_low aneuploid ones (Fig. 1E).

**Table 1:** Essential genes in MAD2_high aneuploid cell lines in CRISPR-CAS9 datasets.

Gene_symbol	Effect_value	*p*-value	log10 *p*-value
ITGA3	-0.171301602	4.24E-08	7.372813671
LEMD2	-0.204123872	7.26E-08	7.138856593
WDR26	-0.241765338	3.54E-07	6.450892207
TP63	-0.173451336	7.08E-07	6.150019366
KIF22	-0.130670319	1.08E-06	5.967583584
PPP6R3	-0.18697065	1.21E-06	5.918129029
MAEA	-0.221636185	1.43E-06	5.844612582
STT3A	-0.168696809	1.53E-06	5.816053384
ILK	-0.262394521	1.85E-06	5.733852398
CRKL	-0.356150021	5.26E-06	5.279215951
HSPA13	-0.16540947	5.30E-06	5.276002326
ZBTB17	-0.176579589	6.79E-06	5.168426561
ERBB2	-0.190045587	7.82E-06	5.106857078
TUBB4B	-0.228012494	8.23E-06	5.084670769
EGFR	-0.23055226	9.75E-06	5.010971093
ADAR	-0.268183725	9.94E-06	5.002710259
NCKAP1	-0.255338573	1.24E-05	4.907191142
GRK2	-0.117642859	1.56E-05	4.807617051
VPS4B	-0.162516019	2.24E-05	4.648845618
COL1A1	-0.103246773	2.71E-05	4.56733432
CHMP7	-0.227388498	3.48E-05	4.458519068
UBR4	-0.308507694	3.50E-05	4.456206416
KLF5	-0.249603864	3.72E-05	4.429575661
WASL	-0.101109642	4.05E-05	4.392609267
PSMB7	-0.315537995	4.51E-05	4.345531256
CNOT9	-0.184473591	5.52E-05	4.25790514
UBE2H	-0.206832618	5.85E-05	4.232590123
PSMD14	-0.253134707	6.77E-05	4.169276253
SOCS3	-0.218455957	7.90E-05	4.102111348
CTNNA1	-0.134465762	9.04E-05	4.043829797
MYRF	-0.109326724	0.00010268	3.988513619
DLG5	-0.127664048	0.000108807	3.963343846
PXN	-0.163150497	0.000110286	3.9574786
PSMB5	-0.45458242	0.000112084	3.950456269
VPS52	-0.220020813	0.00011227	3.949736378
UBA5	-0.185513515	0.000112463	3.948989095
RELA	-0.151483871	0.000120092	3.920485973
PPP1R12A	-0.410212123	0.000126273	3.898688712
COPS4	-0.200485566	0.000131995	3.879441299
GRHL2	-0.125344522	0.000135367	3.868487808
PCNX3	-0.118821656	0.000135996	3.866472731
YPEL5	-0.286120841	0.000161797	3.79102972
EFR3A	-0.214980136	0.000166138	3.779530316
STAMBP	-0.233044103	0.000166178	3.779426019
HGS	-0.212289278	0.000196938	3.705669493
ESRRA	-0.122336242	0.000215054	3.667453353
KIF18A	-0.370834645	0.000218826	3.659900526
ERBB3	-0.109395456	0.000221629	3.654373066
FOSL1	-0.211397771	0.000251289	3.599826919
ARHGEF7	-0.197240016	0.000315136	3.501501415
ARHGAP29	-0.115668237	0.000315171	3.501453554
LIMS1	-0.165655122	0.000414875	3.38208262
BUB1B	-0.203184648	0.000457105	3.339983726
VPS4A	-0.119565094	0.000458297	3.338853116
PIP5K1A	-0.135652013	0.000502611	3.298768154
UBA1	-0.18717339	0.000503718	3.297812479
ACACA	-0.172307059	0.000527955	3.277402767
ACTG1	-0.160795781	0.000541755	3.266197335
PTPN23	-0.221232126	0.000564866	3.248054413
SKA1	-0.171014152	0.000629829	3.20077706
STXBP3	-0.170986385	0.000720987	3.142072536
SARS1	-0.177309694	0.000726439	3.13880114
UFM1	-0.180580151	0.000803049	3.095257746
PKN2	-0.161984588	0.000803121	3.095219005
WTAP	-0.18551805	0.000812367	3.090247644
UBA6	-0.127704637	0.0008205	3.085921345
SLC33A1	-0.141353056	0.0008487	3.071245842
PPP2R1A	-0.296568082	0.000874296	3.058341578
HSP90B1	-0.142571833	0.000917968	3.037172619
SNRPB2	-0.233271397	0.00092314	3.034732313
NUP58	-0.109637565	0.000941219	3.026309487
PARD6B	-0.175355957	0.000962125	3.016768469
CCNE1	-0.181579946	0.000962247	3.016713399
STX4	-0.226443165	0.000966183	3.01494068
MOB4	-0.203384996	0.001031567	2.986502756
RHEB	-0.213306374	0.001045183	2.980807755
MARK2	-0.135365166	0.001178743	2.928580987
DYNLRB1	-0.252453618	0.001210917	2.91688564
ITGB1	-0.157512312	0.001222307	2.912819752
ARF1	-0.157005819	0.001336345	2.874081299
BIRC6	-0.15880782	0.00135345	2.868557637
ALG5	-0.11395965	0.001427025	2.84556828
GRB2	-0.277924997	0.001447779	2.839297636
STRIP1	-0.144246654	0.001618592	2.79086254
KANSL3	-0.200460905	0.001818359	2.740320461
RAC1	-0.227858322	0.001866344	2.72900831
CRK	-0.100566014	0.001922464	2.716141871
CDK2	-0.218820533	0.002121123	2.673434211
TEAD1	-0.124516392	0.002185298	2.660489322
DERL1	-0.101583116	0.002225274	2.652616589
SON	-0.194116396	0.002245057	2.648772576
DDX3X	-0.329372252	0.002431349	2.614152625
EIF1AX	-0.376735711	0.002434267	2.61363172
UFC1	-0.14766641	0.002439824	2.612641478
PPP1R7	-0.155604292	0.002504379	2.601300025
DYNC1LI2	-0.101847766	0.002666867	2.573998611
SNRNP70	-0.148231694	0.002868561	2.542335937
ANLN	-0.149116715	0.002941745	2.53139501
UFL1	-0.123270334	0.003005955	2.522017523
KIF2C	-0.116492905	0.003119803	2.505872823
RBM10	-0.120837767	0.003157	2.500725418
SLC35B2	-0.116646552	0.003296827	2.481903873
SF3B2	-0.163324916	0.003307907	2.48044677
MPRIP	-0.100658587	0.003364618	2.473064181
PRPF31	-0.161152663	0.003396985	2.46890639
TRRAP	-0.16103613	0.003473674	2.459210912
CCDC130	-0.148868888	0.003596862	2.444076265
FASN	-0.128573326	0.003817277	2.418246282
ALG14	-0.1415485	0.004006144	2.397273457
ZNF407	-0.134967841	0.004164063	2.380482695
CDK12	-0.113594512	0.004319943	2.364522031
COPG1	-0.238461069	0.004357131	2.360799397
NUP160	-0.128120574	0.00483827	2.315309868
COPZ1	-0.154226413	0.004863861	2.313018866
HIKESHI	-0.103103901	0.00498078	2.302702657
WBP11	-0.147067553	0.004983204	2.302491312
HECTD1	-0.124176971	0.005247737	2.280027943
JUNB	-0.10518688	0.00532559	2.273632301
TGIF1	-0.102793981	0.005371689	2.269889113
TRAF2	-0.149562433	0.005457826	2.262980294
PAX8	-0.196250306	0.005686079	2.245187092
SLC39A10	-0.109799057	0.005802219	2.236405896
METAP1	-0.137096047	0.005847959	2.232995657
KDM2A	-0.146872154	0.005853663	2.232572253
KCMF1	-0.195264684	0.006079474	2.216133994
RPAP3	-0.132966153	0.006123725	2.2129843
CCDC51	-0.115604539	0.006358621	2.196637079
DBF4	-0.154342822	0.006731994	2.171856252
SBDS	-0.216433335	0.006913341	2.160312049
CENPE	-0.165294862	0.007022179	2.153528084
SRP14	-0.16961429	0.007182816	2.143705272
FAAP20	-0.139944198	0.007491656	2.125422182
PPP1CA	-0.124436287	0.007754575	2.110441975
CLTC	-0.189730946	0.007766275	2.10978723
TRIP13	-0.123296885	0.008557466	2.06765481
RANBP2	-0.111456393	0.008720215	2.059472825
CHMP3	-0.16428162	0.009160577	2.038077165
HNF1A	-0.105820347	0.009206882	2.035887433
ODR4	-0.10931581	0.009726583	2.012039706
SF3B6	-0.167450606	0.009792971	2.009085525
CSNK2B	-0.157925655	0.009840845	2.006967588
H2BC11	0.239954173	8.34E-06	5.078574503
TINF2	0.329623825	9.18E-06	5.037368616
H2AZ1	0.259956429	1.03E-05	4.989029277
MZF1	0.12904724	2.48E-05	4.60507321
UPF3A	0.163989132	4.73E-05	4.325091831
PPM1D	0.223147894	6.81E-05	4.166578358
POT1	0.242404079	7.18E-05	4.143821643
ESF1	0.191401154	0.000106291	3.973502231
PGBD2	0.10269345	0.000110382	3.957103406
NIP7	0.207649709	0.00015262	3.816389547
DDX27	0.171900292	0.000203379	3.691693097
MDM4	0.232264205	0.000255912	3.591910019
BRIP1	0.145337334	0.00025891	3.586851489
WDR74	0.197157411	0.00026874	3.570667106
DROSHA	0.142491135	0.000299183	3.524063445
ZFR	0.118761556	0.000369554	3.432322328
NIPBL	0.222629729	0.000396434	3.401829166
CYC1	0.207911632	0.000412743	3.384320425
RPL22L1	0.159199282	0.000429328	3.36721061
COX20	0.114920078	0.000437246	3.359273803
ATP1B3	0.20599716	0.000444952	3.351687223
PAGR1	0.178061802	0.00053909	3.268338463
CCDC115	0.204457963	0.000541698	3.266242554
WRN	0.263578363	0.00056619	3.247038007
ACD	0.127580981	0.000621318	3.206686021
DCLRE1B	0.228539939	0.000631128	3.199882643
CENPO	0.12234167	0.000646258	3.189593788
CDAN1	0.28240285	0.000649088	3.18769672
SINHCAF	0.162328078	0.000684035	3.16492143
MDM2	0.29102423	0.000827179	3.082400322
USP7	0.214426482	0.000875659	3.057664825
UBE2D3	0.218004533	0.000876497	3.057249487
POLR3K	0.190564282	0.001001782	2.999226676
MAU2	0.187813655	0.001070549	2.970393636
GCSH	0.13603091	0.001114286	2.9530033
EIF6	0.149301604	0.001272541	2.895328127
FANCA	0.123802289	0.001297291	2.88696259
FNTA	0.171971563	0.001428904	2.844997091
SDHA	0.140827034	0.001536951	2.813340034
C8orf33	0.125680466	0.001552924	2.808849673
FANCL	0.105959424	0.001577216	2.802108836
WDR75	0.183798789	0.001616515	2.791420237
SPATA5L1	0.209056131	0.001818551	2.740274405
EZH2	0.135728682	0.001951271	2.709682409
TAF4	0.10452218	0.001964796	2.706682525
HEATR1	0.171098378	0.001997707	2.699468176
PFN1	0.187995344	0.002148839	2.667796117
DDX51	0.152107935	0.002159732	2.6656002
EXOC8	0.13136099	0.002258415	2.646196249
DNAJC9	0.299827519	0.002332503	2.632177754
DPAGT1	0.241155986	0.002373439	2.624621953
PAXIP1	0.140780114	0.002635244	2.579179115
FANCI	0.125817385	0.003048152	2.515963396
RBM39	0.154018389	0.003239244	2.489556383
DHODH	0.195961106	0.003283528	2.483659324
COMMD8	0.103429898	0.003337764	2.476544352
YARS2	0.213344535	0.003747163	2.426297436
EP300	0.19376633	0.00376749	2.423947855
STK11	0.164763065	0.00378451	2.421990337
NAMPT	0.205206885	0.00395068	2.403328167
EIF2AK4	0.118639881	0.004037482	2.393889395
TTF2	0.136760272	0.004081819	2.389146253
CINP	0.182674059	0.004084922	2.388816245
CENPP	0.11884938	0.004227506	2.373915758
NOL9	0.137859481	0.004276412	2.368920413
EXOC4	0.100371614	0.004398311	2.356714022
PDAP1	0.165267914	0.004506327	2.346177249
ISG20L2	0.110934444	0.004825859	2.316425368
LAS1L	0.157739364	0.004935292	2.306687109
RPL27A	0.145588	0.004960525	2.304472353
IKZF1	0.10149071	0.005207458	2.283374262
RAD1	0.124180827	0.005220149	2.282317067
KNTC1	0.110446624	0.005313509	2.274618552
RPL8	0.120831655	0.005449574	2.263637434
C15orf41	0.149017682	0.00547116	2.261920554
ACTR8	0.141662735	0.005611988	2.25088329
RPS5	0.11116287	0.005712788	2.243151866
EEF2KMT	0.106601278	0.005721225	2.242510996
NOP9	0.170848868	0.005893616	2.22961813
LIAS	0.174943522	0.006147439	2.211305797
ZWILCH	0.10048423	0.006149904	2.211131644
H2AC4	0.10857325	0.006150287	2.211104589
PET117	0.124855008	0.00627487	2.202395265
DLD	0.154109885	0.006306706	2.200197427
TIMELESS	0.140039655	0.006408197	2.193264153
AAMP	0.152386571	0.006554237	2.183477832
RPS3	0.13701998	0.006583509	2.181542595
LSG1	0.122068025	0.006660178	2.176514156
COA6	0.118462101	0.006828055	2.165702973
TRIM28	0.108995824	0.006972232	2.156628182
RNASEH2A	0.118071157	0.007106817	2.14832484
SMC3	0.128812296	0.007862957	2.104414089
H2AC20	0.186524168	0.007998649	2.096983352
TMEM189	0.104578931	0.008550352	2.068015998
COX6C	0.100541072	0.009238124	2.034416216
ACTR6	0.129575108	0.009336308	2.029824824
RBSN	0.130270387	0.009352466	2.029073878
ALG2	0.180257327	0.009699713	2.013241105
PCBP1	0.186431443	0.009780921	2.00962026
GTF2F1	-0.206135662	8.37E-08	7.077513331
LARP4	-0.136821387	6.17E-06	5.209927643
NUP58	-0.164645201	6.86E-06	5.163416317
PSMB7	-0.344861902	7.30E-06	5.136540072
ERBB3	-0.113769097	7.90E-06	5.102562703
PSMD14	-0.280851031	8.69E-06	5.060818577
COPS4	-0.217285692	1.16E-05	4.936231157
LEMD2	-0.185722988	1.51E-05	4.820821745
UBA6	-0.160165018	1.59E-05	4.798953496
ADAR	-0.254536364	1.91E-05	4.71860227
WWTR1	-0.213421585	2.61E-05	4.582792244
TROAP	-0.119337398	3.71E-05	4.430682595
PSMB5	-0.479879068	5.59E-05	4.252925838
SOX9	-0.175181698	6.09E-05	4.215656385
CWC25	-0.145198472	7.32E-05	4.135505215
POLR2E	-0.214324848	0.000127389	3.894869094
HSP90B1	-0.162929552	0.00013055	3.884224494
CAND1	-0.152112861	0.000132699	3.877131379
ASPM	-0.126783723	0.000164028	3.7850824
PPP1R12A	-0.377079408	0.000240862	3.618231374
INTS2	-0.179540683	0.000264554	3.577485511
ASIC1	-0.100139285	0.000274526	3.561416845
ILK	-0.189019291	0.000278878	3.554585469
USP14	-0.114622823	0.000313981	3.50309702
FCHO2	-0.125084104	0.000331843	3.479066893
ELAVL1	-0.140209882	0.000332862	3.477736057
RBM5	-0.125196644	0.000369473	3.432417579
PMPCA	-0.208100852	0.000380716	3.419398382
ALG11	-0.242489038	0.000415307	3.381630652
FBXO42	-0.167197309	0.000454342	3.342616641
MED21	-0.14079705	0.000481873	3.317067496
WTAP	-0.186051776	0.000489649	3.31011543
SCYL1	-0.134799137	0.000557883	3.253456818
TUBGCP4	-0.182843106	0.000568919	3.244949206
PSMD13	-0.165431979	0.000597305	3.223803842
BIRC6	-0.166860626	0.000617545	3.209331359
DDX20	-0.16222063	0.000678198	3.168643496
KCTD10	-0.202778922	0.000699737	3.155064979
RNH1	-0.10501024	0.000700125	3.15482423
PAX3	-0.108472866	0.000722206	3.141338769
PSMA6	-0.161856569	0.00080614	3.093589722
TEAD1	-0.14120296	0.00084374	3.073791392
KRT18	-0.155497966	0.000875111	3.057937079
MED8	-0.176003373	0.000943403	3.025302743
EAF1	-0.227547897	0.000959389	3.018005206
PDZK1	-0.105250696	0.001007825	2.996614748
PPRC1	-0.133129784	0.001036271	2.984526502
COPB2	-0.209293566	0.001132601	2.945922939
INTS13	-0.129326784	0.001201125	2.920411631
C7orf26	-0.153727898	0.001302613	2.885184751
SOX10	-0.146313795	0.001317091	2.880384172
SNUPN	-0.191405084	0.001343878	2.871640265
FOXM1	-0.114840029	0.00140415	2.852586379
TFIP11	-0.150075124	0.001569812	2.804152493
MYO1H	-0.108776792	0.001601545	2.795460986
UBR4	-0.225961269	0.001620973	2.790224166
TUBA1B	-0.19713891	0.001632079	2.787258783
PRR13	-0.101213253	0.001660074	2.779872621
TP53	-0.242657246	0.001690092	2.772089747
ODR4	-0.129644239	0.001692193	2.771550165
SRSF10	-0.168047716	0.001766925	2.752781923
SYMPK	-0.147500178	0.001776948	2.750325159
WDR73	-0.197827531	0.001788556	2.747497359
CHMP7	-0.170918791	0.001820239	2.739871637
SEC13	-0.152360869	0.0018395	2.735300255
SGO1	-0.243262796	0.001958533	2.708069161
STXBP3	-0.162511457	0.002023664	2.69386168
GMNN	-0.198901349	0.002027326	2.693076338
LUC7L2	-0.106177574	0.002078298	2.68229225
TLN1	-0.118727781	0.002169165	2.663707501
LSM8	-0.188876788	0.002223094	2.653042102
UFM1	-0.164325063	0.002358569	2.627351497
CDK2	-0.194311732	0.002447098	2.611348624
ECT2	-0.185093845	0.002552097	2.593102857
NAPG	-0.208854356	0.002608412	2.583623794
DYNLRB1	-0.230213027	0.002705899	2.567688403
PPP1CA	-0.138937165	0.002718176	2.565722394
CRKL	-0.228529363	0.002796244	2.553425004
FASN	-0.143918553	0.002884258	2.539965841
CWC22	-0.177888	0.003069008	2.513001965
BORA	-0.128945693	0.003086825	2.510487985
RAN	-0.130355935	0.003114233	2.506648892
ITGAV	-0.242592351	0.003129886	2.504471508
GOSR2	-0.168862771	0.003178405	2.497790821
PRDX1	-0.115025226	0.003458851	2.461068184
PSMC4	-0.152568224	0.003494973	2.456556176
SMU1	-0.139458123	0.003961369	2.402154687
GLMN	-0.105315652	0.004188776	2.377912829
RACGAP1	-0.173779896	0.004297677	2.366766256
HECTD1	-0.116068725	0.004399757	2.356571318
TOE1	-0.168193522	0.004656348	2.331954572
NUP62	-0.127296101	0.004670869	2.330602307
UFC1	-0.136590287	0.004922096	2.307849882
KCMF1	-0.199521672	0.005055856	2.296205343
MAEA	-0.116638032	0.005279526	2.277405068
FDX2	-0.123724722	0.00529485	2.276146374
IER3IP1	-0.116672935	0.005313243	2.274640316
PXN	-0.109736549	0.005623034	2.25002926
SFSWAP	-0.115568448	0.005629886	2.249500375
ZC3H13	-0.118740756	0.005737027	2.24131314
PSMD8	-0.136258752	0.006021583	2.220289331
SKA3	-0.158699529	0.006176757	2.209239468
MCRS1	-0.111364321	0.006515853	2.186028728
RANBP2	-0.121725114	0.006733975	2.171728523
UBA5	-0.130384063	0.006933513	2.15904668
NCBP1	-0.135697229	0.007018444	2.153759132
UPF1	-0.143821976	0.007039373	2.152466
ERBB2	-0.107042337	0.007105704	2.148392916
COPS3	-0.133811733	0.007522028	2.123665038
NAA50	-0.159155551	0.008330043	2.079352744
CCT3	-0.144186821	0.008606298	2.065183624
SRP54	-0.122698017	0.009618802	2.016879007
TRAPPC3	-0.162606365	0.009722891	2.012204572
PDCL3	-0.114782126	0.009762463	2.010440605
COPZ1	-0.13918989	0.00986168	2.006049079

FOXM1 controls cell cycle-related gene expression and regulates chromosome stability [27, 28]. To understand if the requirement of FOXM1 is associated with aneuploidy or with MAD2 levels, we examined essential genes of MAD2_low aneuploid cells as well as those in MAD2_high euploid cells. In both cases, *FOXM1* was not found in either CRISPR or RNAi datasets (Table 2). Moreover, CRISPR and RNAi datasets analyses indicated that MAD2_high euploid cells are not sensitive to FOXM1 inhibition (Supplementary Fig. 1D). Since *FOXM1* mRNA expression is similar in MAD2_high euploid and aneuploid cells (Supplementary Fig. 1C), we hypothesized that FOXM1 essentiality might be related to MAD2 expression and aneuploidy status.

**Table 2:** Essential genes in MAD2_high aneuploid cell lines in RNAi screens.

Gene_symbol	Effect_value	*p*-value	log10 *p*-value
EGFR	-0.308578203	1.51E-05	4.820025863
APLP1	-0.23852302	4.27E-05	4.370026535
VPS28	-0.310032615	5.16E-05	4.287644716
PI4KAP1	-0.238541055	7.65E-05	4.116088832
ANAPC4	-0.201378928	0.00012971	3.887026088
MRPL34	-0.276448135	9.64E-05	4.015812547
RCAN2	-0.268963692	9.89E-05	4.004964582
ZNF79	-0.208024472	0.000234149	3.630507031
SST	-0.31239922	0.000276512	3.558286048
SART3	-0.28228609	0.000388177	3.410969653
ADAR	-0.200744216	0.00073785	3.132032076
ARL14EP	-0.237539553	0.000569577	3.244447766
SMC1A	-0.282926945	0.001029789	2.987251886
RPF1	-0.201916831	0.000839813	3.075817418
PGS1	-0.200095346	0.001230349	2.909971561
RAPGEF1	-0.213022516	0.001239584	2.906724171
UBE2L3	-0.204999533	0.001264672	2.898022248
USPL1	-0.232382875	0.001290869	2.889117929
FUBP1	-0.214308276	0.001338524	2.873373918
INHA	-0.220682393	0.001771312	2.751705028
DSTNP2	-0.29206211	0.001518297	2.81864331
ZNF318	-0.245551147	0.001806694	2.74311528
UBC	-0.421159871	0.002147215	2.668124465
FSCN1	-0.223634796	0.002137261	2.670142383
MTDH	-0.219000993	0.002178337	2.661874984
RPN2	-0.411459309	0.002239366	2.649875002
TARS2	-0.223181218	0.002731781	2.563554134
CENPT	-0.331432068	0.002432018	2.614033261
UBA1	-0.213930275	0.003335688	2.476814571
LRATD1	-0.236835891	0.002880242	2.540570988
HAUS1	-0.224064421	0.003525742	2.452749478
TP53AIP1	-0.204523372	0.003036952	2.51756204
C5orf64	-0.234809758	0.0039892	2.399114216
BUB1B	-0.223459068	0.00432006	2.36451025
SPC25	-0.230702889	0.004246631	2.371955471
INTS7	-0.263138641	0.004445474	2.352081948
TSG101	-0.202908504	0.005464938	2.262414799
PSMD6	-0.251494278	0.00602576	2.219988148
MAD2L1	-0.27438668	0.007375051	2.132234968
HHLA1	-0.205759763	0.007552781	2.121893097
C10orf120	-0.205647495	0.008442886	2.073509074
OR11H6	0.323607598	2.26E-05	4.645673593
RPS21	0.362999893	0.000207412	3.683165395
PLK4	0.20581321	0.000379124	3.421219093
MDM4	0.40455511	0.000834803	3.078415862
MGST3	0.232868614	0.000511418	3.291223678
LINC00326	0.290331754	0.000523832	3.280808258
BCCIP	0.243594823	0.00156925	2.804307949
RPS15A	0.290422498	0.001835186	2.736319989
RPL38	0.287385607	0.001682885	2.773945568
BIRC5	0.26940394	0.002848195	2.545430353
MTUS2	0.205307588	0.002582497	2.587960254
PSCA	0.236794954	0.003164556	2.499687232
APLF	0.203430665	0.004106636	2.386513843
BOP1	0.210500144	0.005650307	2.247927978
EIF3H	0.228717862	0.007044345	2.152159383
MDM2	0.244448163	0.007229229	2.140908023
TMEM140	0.200440535	0.006109372	2.214003458
AAMP	0.254828868	0.008693094	2.060825637
RAE1	0.21367536	0.008821265	2.054469153
WDR43	0.277079671	0.008840093	2.05354318
RPS16	0.243133409	0.00919962	2.036230108
WDR61	0.208403634	0.009450795	2.024531675
FOXM1	-0.2097178923296	1.01E-05	4.995013071
LPAR6	-0.2079895135020	5.21E-05	4.283041249
FSCN3	-0.2847312925564	7.03E-05	4.153305844
CDC27	-0.2971428828023	0.000312215	3.505546661
MSH5	-0.2011334620033	0.000391493	3.407275541
CDK1	-0.3408037501756	0.000512553	3.290261608
CCHCR1	-0.2635537233277	0.000342906	3.464824687
ZNF318	-0.2711877263380	0.000404133	3.393475161
PPP1R12A	-0.2634983471912	0.000913836	3.039131625
ACOT8	-0.2411169237788	0.000922371	3.035094221
FBXO38	-0.2266372104735	0.000593844	3.226327928
VPS36	-0.2188040307346	0.000611075	3.213905154
NOP2	-0.3243509088046	0.000742786	3.129136487
NAA50	-0.2575658499608	0.001443196	2.840674674
OR5M10	-0.2167254601240	0.002225402	2.65259146
TMEM225	-0.2058964254804	0.001720826	2.764262933
KRT9	-0.2150193086951	0.001821093	2.739667815
CHMP2B	-0.2043083983964	0.001846672	2.733610307
INTS10	-0.2186960788879	0.001870535	2.728034136
AHSP	-0.2169071582377	0.002110482	2.675618301
ATP6V1B2	-0.2218201575161	0.003309794	2.480199031
GLIPR1	-0.2114206414148	0.002432037	2.614029735
MAU2	-0.2353486352053	0.002628525	2.580287925
BUB1B	-0.2318257926958	0.003624869	2.440707697
OTULIN	-0.2326159296883	0.002687231	2.570695
ZNF207	-0.2163062876973	0.00391905	2.406819215
PCNP	-0.2496381276210	0.003025172	2.519249971
DENND4B	-0.2190534261805	0.00277615	2.556557066
MZB1	-0.2459989060438	0.002820203	2.549719699
UBA1	-0.2101776373209	0.004371594	2.359360175
UBL5	-0.2181761244744	0.005134203	2.289526977
TTLL11	-0.2240823145353	0.004297638	2.366770122
C6orf62	-0.2289944039338	0.004831949	2.315877696
CXCR2P1	-0.2255134047974	0.008340458	2.078810087
JPT1	-0.2555496082810	0.008109553	2.091003089
CEMIP	-0.2197280291959	0.008432146	2.07406186
RCC1	-0.2100687839592	0.009817042	2.00801937
LINC01602	-0.2179758300006	0.008000508	2.096882442
C12orf43	-0.7242970232442	0.002444749	2.611765708

To further confirm if FOXM1 is crucial in MAD2_high aneuploid cells, we examined two different drug screening datasets between MAD2_high aneuploid cell lines and euploid cell lines (Fig. 1A). Drug sensitivity data (AUC) suggested that MAD2_high aneuploid cells were more sensitive than euploid cells to the FOXM1 inhibitor Thiostrepton (Supplementary Fig. 1E). Moreover, FOXM1 has been described to be a target for the proteasome inhibitors Bortezomib, Delanzomib and Oprozomib [29, 30]. Analysis of PRISM drug dose-level dataset indicated that MAD2_high aneuploid cells were more sensitive to proteasome inhibitors than euploid cells (Supplementary Fig. 1F). Altogether, these results suggest that aneuploid cells with high MAD2 levels are more vulnerable to FOXM1 inhibition.

### FOXM1 inhibition decreases mitotic fidelity in short-term MAD2 overexpressing cells

**Fig. 2:**
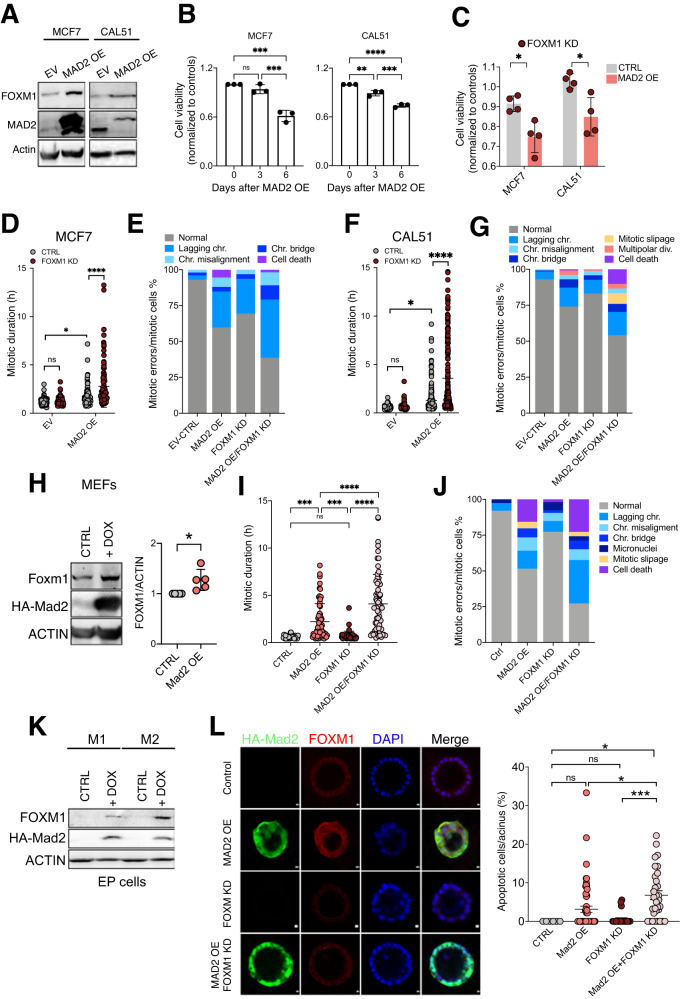
FOXM1 inhibition is detrimental to cell fidelity of Mad2 overexpressing cells. **A** Western blots of FOXM1 and MAD2 in human breast cell lines infected with an empty vector (EV) or a Dox-inducible MAD2 expressing vector (MAD2 OE) after dox administration for 6 days. ACTIN was used as a loading control. Three biological replicates were analyzed. **B** Cell viability of each human cell line after dox treatment for 3 or 6 days. Each dox-treated cell line was normalized to the untreated one. MCF7: ***P < 0.0005; MDA-MB-231: ***P < 0.0001; CAL51: ****P < 0.0001, ***P = 0.0004, **P = 0.0027; MCF10A: **P < 0.008, *P = 0.0359, One-way ANOVA. Each dot is a biological replicate. **C** Cell viability of human cell lines after MAD2 overexpression and *FOXM1* knockdown by siRNA for 6 days. Values of each cell line were normalized to those of each EV group. Each dot is a biological replicate. MCF7: *P = 0.0228; CAL51: *P = 0.0124; Two-way ANOVA. **D** Mitotic duration of MCF7 cells with or without MAD2 overexpression and si*FOXM1* after 3 days. *P = 0.01, ****P < 00001; Two-way ANOVA. (EV, 100 cells; MAD2 OE, 92 cells; FOXM1 KD, 95 cells; MAD2 OE/FOXM1 KD, 119 cells in at least 3 independent experiments). **E** Cell fate of MCF7 cells represented as the incidence of each cell fate/total number of mitotic cells. (EV, 100 cells; MAD2 OE, 92 cells; *FOXM1* KD, 95 cells; MAD2 OE/FOXM1 KD, 119 cells in at least 3 independent experiments). **F** Mitotic duration of CAL51 cells with or without MAD2 overexpression and si*FOXM1* after 3 days. *P = 0.02, ****P < 00001; Two-way ANOVA. (EV, 133 cells; MAD2 OE, 237 cells; FOXM1 KD, 95 cells; MAD2 OE/FOXM1 KD, 234 cells, in at least 3 independent experiments). **G** Cell fate of CAL51cells represented as the incidence of each cell fate/total number of mitotic cells. (EV, 133 cells; MAD2 OE, 237 cells; FOXM1 KD, 95 cells; MAD2 OE/FOXM1 KD, 234 cells, in at least 3 independent experiments). **H** Representative western blot of *KH2-HA-Mad2/Rosa26-rtTA* MEFs and quantification in 5 MEFs lines, that were either not induced (CTRL) or on Dox for 30 h. Actin was used as loading control. FOXM1/ACTIN levels were normalized to control groups. *P = 0.0193; Unpaired *t*-test. 5 biological replicates were analyzed. **I** Mitotic duration of MEFs with or without MAD2 overexpression and si*FOXM1* (CTRL, 38 cells; MAD2 OE, 64 cells; FOXM1 KD, 53 cells; MAD2 OE/FOXM1 KD, 66 cells in 3 independent movies). ***P = 0.0007; ****P = < 0.0001. One-way ANOVA. **J** Cell fate of MEFs with or without MAD2 overexpression and si*FOXM1* (CTRL, 38 cells; MAD2 OE, 64 cells; FOXM1 KD, 53 cells; MAD2 OE/FOXM1 KD, 66 cells in 5 MEFs cell lines). **K** Representative western blot of FOXM1 and HA-Mad2 in EP cells from two *TetO-Mad2/MMTV-rtTA* Dox-inducible transgenic mice (M1 and M2) without DOX (CTRL) and with DOX. Actin was used as loading control. **L** Representative immunofluorescence images of HA, FOXM1 and DAPI on fixed mammary spheroid cultures after 36 h on Dox or/and FOXM1 inhibition by RCM-1. Scale bar: 10 μm. Percentage of apoptotic cells per acinus was calculated as apoptotic cells/total cells in each acinus (CTRL, 6 acini; MAD2 OE, 56 acini; FOXM1 KD, 24 acini; MAD2 OE/FOXM1 KD, 32 acini). ***P = 0.0004, *P = 0.0329 and 0.0178; One-way ANOVA. For original and additional wbs see Supplemental Material. Raw data for D,E,F,G, I J is included in Supplemental Tables.

Dysregulation of FOXM1 disrupts mitosis and increases CIN [31, 32]. To study the impact of FOXM1 inhibition on mitosis in MAD2-overexpressing cells (MAD2 OE), we generated doxycycline (Dox) inducible human MAD2-expressing breast cancer cell lines (MCF7, CAL51, MDA-MB-231, and MCF10A). MAD2 overexpression resulted in increased FOXM1 levels after 6 days (Fig. 2A and Supplementary Fig. 2A and 2B) and reduced cell viability in all cell lines over time (Fig. 2B and Supplementary Fig. 2C). We then used siRNAs against *FOXM1* in these cell lines, infected with an empty vector or with the Dox inducible MAD2 vector and monitored the consequences of FOXM1-downregulation (FOXM1 KD) 6 days after induction. Cell viability was significantly reduced in MAD2 OE cells after FOXM1 KD compared to the inhibition of FOXM1 alone (Fig. 2C and Supplementary Fig. 2D).

We then selected two of these cell lines, one unstable breast cancer cell line, MCF7 and one diploid cell line, CAL51, and performed live cell imaging to study if mitotic defects or mitotic timing were contributing to the reduced viability. Time-lapse microscopy revealed that FOXM1 KD prolonged mitotic duration in MAD2 expressing MCF7 and CAL51 cells but not in the ones infected with an empty vector (Fig. 2D and F). In addition, we found a significant increase in the number of mitotic errors when FOXM1 was downregulated in both cell lines (Fig. 2E and G and Supplementary Fig. 2E, F). Strikingly, MAD2-expressing cell lines had the highest rates of mitotic errors after FOXM1 depletion, suggesting that FOXM1 depletion not only affected mitotic duration but also increased the incidence of lagging chromosomes and other mitotic errors when MAD2 was overexpressed.

We next generated mouse embryonic fibroblasts (MEFs) from *KH2-Mad2/Rosa26-rtTA* Dox inducible transgenic mice [13]. Induction of MAD2 after 30 h on Dox led to higher FOXM1 protein levels (Fig. 2H). We previously published that overexpression of MAD2 causes mitotic delay and CIN in these cells [12]. Inhibition of FOXM1 had no significant effect on mitotic duration in wild-type MEFs while it induced a mild mitotic delay in MAD2-overexpressing cells (Fig. 2I). In addition, we observed increased number of mitotic errors upon MAD2 OE or FOXM1 KD in cells that were further increased in the combination of both. Interestingly, an increase in cell death and mitotic errors was observed in MAD2 OE cells after FOXM1 KD (Fig. 2J). These results indicate a causal link between MAD2 induced CIN and FOXM1 dependency. Altogether, our results show that whereas normal cells tolerate the downregulation of FOXM1, it results in prolonged mitosis and increased CIN levels in MAD2 OE cells.

Finally, we used mammary epithelial (EP) cells from our *TetO-HA-Mad2/MMTV-rtTA* doxycycline-inducible transgenic mice (M) where short-term MAD2 overexpression has been shown to induce mitotic arrest and cell death [13]. Consistent with our observations in MAD2 OE MEFs, we observed high FOXM1 protein levels after MAD2 induction in EP cells (Fig. 2K) and immunofluorescence staining confirmed that tipically cells with HA-MAD2 overexpression showed FOXM1 upregulation (Supplementary Fig. 2G). We then measured the effect of FOXM1 downregulation using the RCM-1 inhibitor on 3D cultures grown from the EP of M mice [33]. MAD2 overexpression led to the accumulation of dying cells with no expression of FOXM1, while cells at the rim of these spheres retained expression of HA-MAD2 and FOXM1. Additional inhibition of FOXM1 in MAD2 overexpressing organoids led to an increase in the number of apoptotic cells and the shrinkage and condensation of the acinar structures, while FOXM1 KD in normal EP cells did not increase apoptotic cells (Fig. 2L). Thus, FOXM1 KD is detrimental in human breast cancer cells and mouse cells that express high levels of MAD2.

### FOXM1 is required for the fitness of high MAD2 unstable cells

**Fig. 3:**
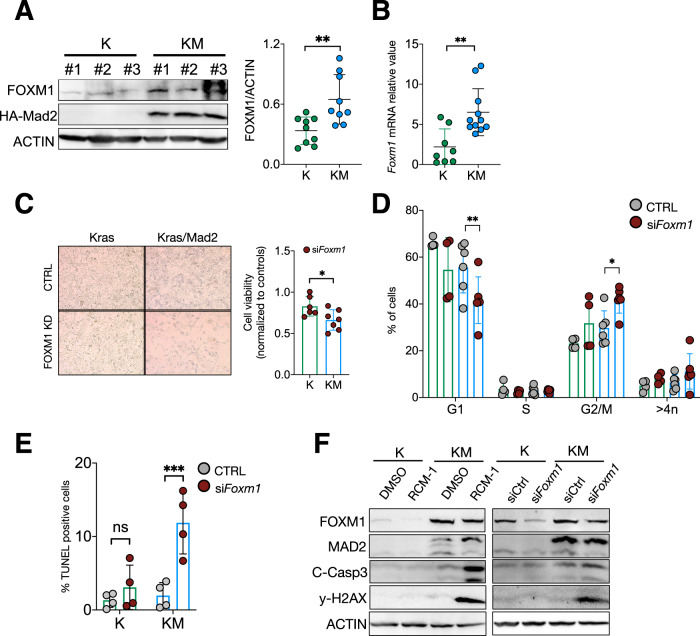
FOXM1 knockdown impairs fitness of MAD2-overexpressing cells. **A** Representative western blot and quantification of FOXM1 and HA-Mad2 in Kras (K1, K2, K3) and Kras/Mad2 (KM1, KM2, KM3) breast tumor cells. **P = 0.0043; Unpaired *t-*test. (*n* = 9 K and 9 KM tumors were analyzed and measured). **B** Quantitative RT-PCR of mouse *Foxm1* expression in mammary tumors from K and KM animals (*n* = 8 K and *n* = 11 KM tumors). *P = 0.0338; Unpaired *t*-test. **C** Representative pictures (left) and relative cell viability (right) of K and KM tumor cells after si*Foxm1* for 6 days (*n* = 6 K and *n* = 7 KM tumors with si*Foxm1*). Cell viability was normalized to untreated cells. *P = 0.0313; Unpaired *t-*test. **D** DNA content analysis of K and KM breast tumor cells after si*Foxm1* for 6 days represented as the percentage of cells in each cell cycle phase. (*n* = 4 K and *n* = 6 KM tumors were analyzed). **P = 0.001, *P = 0.0304; Two-way ANOVA. **E** Percentage of TUNEL positive cells in K and KM tumor cells 6 days after *Foxm1* knockdown (KD). (*n* = 4 K and 4KM tumors were analyzed). ***P = 0.0006; Two-way ANOVA. **F** Western blots of FOXM1, HA-Mad2, Cleaved-caspase3 (C-Casp3) and gama-H2AX in K and KM tumor cells after RCM-1 treatment or si*Foxm1* for 6 days, performed in two biological replicates. ACTIN was used as a loading control. For original and additional wbs see Supplemental Material.

MAD2 overexpression in *Kras*-induced breast tumors leads to the formation of chromosomally unstable tumors that retained MAD2 expression [13], suggesting that these MAD2 tumor cells adapt over time to high MAD2 levels. To investigate, whether Foxm1 can play a role in this adaptation process, we asked if long-term induction of MAD2 also resulted in increased FOXM1 levels. We harvested breast tumor cells from *TetO-Kras/MMTV-rtTA* transgenic mice (K) and *TetO-Kras/TetO-Mad2/MMTV-rtTA* mice (KM) and observed higher *Foxm1* mRNA and protein levels in KM compared to K tumor cells (Fig. 3A, B). To explore the transcription profiling of tumor cells, RNA sequencing data from K and KM tumors was analyzed [24]. Gene set enrichment analysis (GSEA) revealed downregulation in pathways related to APC/C:CDH1 function, chromosomal segregation and anti-apoptotic signaling in KM tumors when compared to K tumors (Supplementary Fig. 3A). These results suggest that long-term MAD2 overexpression might lead to a dysfunctional regulation of mitosis and apoptosis in mouse breast tumor cells. To understand the consequences of FOXM1 inhibition after long-term MAD2 expression, we used siRNA to knockdown *Foxm1* in K and KM tumor cells. si*Foxm1* for 6 days in KM cells resulted in a significantly reduced cell viability (Fig. 3C), the accumulation of G2/M cells (Fig. 3D) and increased TUNEL-positive cells (Fig. 3E) when compared to non-treated ones, while inhibition of *Foxm1* in K cells had no effect. Moreover, treatment with the RCM-1 inhibitor or si*Foxm1* led to increased levels of Cleaved Caspase-3 and Gamma-H2AX (γ-H2AX) in KM cells (Fig. 3F). Although total inhibition of FOXM1 was not achieved in KM cells, we still observed that KM unstable cells are significantly more vulnerable than K cells to the downregulation of FOXM1.

Finally, we overexpressed MAD2 in MCF7 cells for a long time (20 days) and analyzed the effect of inhibiting FOXM1. si*FOXM1* led to severe mitotic arrest in MAD2 OE cells (Supplementary Fig. 3B), which further suggests an essential role of FOXM1 in mitotic segregation. We also noticed higher γ-H2AX in both parental and MAD2 OE MCF7 cell lines after FOXM1 inhibition, indicating a role of FOXM1 in regulating DNA damage [34]. Increased C-caspase3 levels in MAD2 OE cells with si*FOXM1* illustrated that MAD2-expressing cells still rely on FOXM1 for mitotic exit and survival (Supplementary Fig. 3C). Collectively, these data suggest that FOXM1 is critical for the viability of CIN cells induced by MAD2 overexpression.

### FOXM1 overexpression preserves mitotic fidelity in MAD2 overexpressing human breast cancer cell lines

**Fig. 4:**
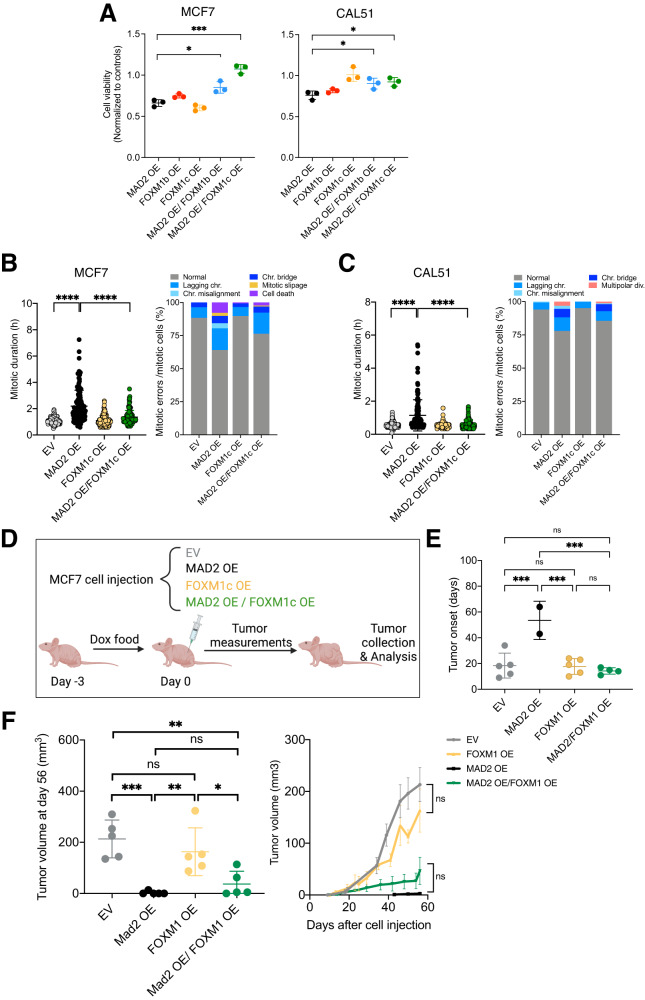
FOXM1 overexpression rescues cell viability and preserves mitotic fidelity in MAD2 overexpressing cells. **A** Cell viability of MCF7 and CAL51 cells after MAD2 or/and FOXM1 overexpression for 6 days. Values were normalized to that of each EV group (*n* = 3 biological replicates). MCF7: ***P = 0.0002, *P = 0.011; CAL51: *P = 0.0452, *P = 0.0225; Ordinary one-way ANOVA. **B** Mitotic duration and cell fate of MCF7 overexpressing MAD2 or/and FOXM1 for 3 days. Mitotic duration was considered from nuclear envelope breakdown until anaphase. ****P < 0001; Ordinary one-way ANOVA. Percentage of cell fate was calculated as the incidence of each cell fate/total number of mitotic cells. (EV, 112 cells; MAD2 OE, 128 cells; FOXM1c OE, 147 cells; MAD2 OE/FOXM1c OE, 130 cells). **C** Mitotic duration and cell fate in CAL51 cells with MAD2 or/and FOXM1 overexpression after 3 days. ****P < 0001; Ordinary one-way ANOVA. Percentage of cell fate was calculated as the incidence of each cell fate/total number of mitotic cells. (EV, 152 cells; MAD2 OE, 127 cells; FOXM1c OE, 123 cells; MAD2 OE/FOXM1c OE, 152 cells). Time lapse microscopy was performed in a minimum of 3 biological replicates. **D** Schematic showing MCF7 cells injected into nude mice that were fed with doxycycline 3 days before injection (*n* = 5 mice per group). **E** Tumor onset recorded as the first day after which a palpable tumor was detected. ***P < 0.001; Ordinary one-way ANOVA. Each dot represents a tumor. **F** Tumor volumes at day 56 after cell injection on the left. ***P < 0.001, **P = 0.0033, *P = 0.0182; Ordinary one-way ANOVA. Tumor volumes overtime on the right. Unpaired *t*-test was applied to compare tumor volumes at day 56. Raw data for B,C is included in Supplemental Tables.

To test whether FOXM1 plays a role in MAD2 tolerance in human cell, we infected MAD2-expressing human breast cancer cell lines with either an empty vector (EV) or with doxycycline-inducible human FOXM1 lentiviral vector (expressing FOXM1b or FOXM1c isoforms). Overexpression of FOXM1 in these cell lines showed similar viability compared to cells infected with the EV six days after induction. However, overexpression of FOXM1 significantly decreased the lethality of MAD2-expressing cells (Fig. 4A) and decreased the percentage of TUNEL-positive MCF7 cells (Supplemental Fig. 4A), suggesting that high FOXM1 levels can rescue MAD2-induced defective cell fitness. Since FOXM1 regulates cell cycle progression as well as chromosome segregation [35], we next sought to understand whether FOXM1 upregulation interferes with MAD2-mediated mitotic arrest and CIN in these cell lines. We performed live-cell imaging of MCF7 and CAL51 cells with or without MAD2 OE and FOXM1 OE. The overexpression of FOXM1 alone did not affect mitotic duration or mitotic errors when compared to EV cells. However, cells overexpressing MAD2 showed a significant decrease in mitotic time and reduced mitotic errors when FOXM1 was overexpressed at the same time (Fig. 4B, C). These results indicate that upregulation of FOXM1 facilitates mitotic exit in MAD2-expressing cells, allowing proper chromosome segregation and consequently, reducing MAD2-induced CIN.

MAD2 overexpression suppresses Aurora B activity, resulting in hyperstable microtubule-kinetochore attachments [36]. We observed that AURORA B expression was suppressed during mitosis in MAD2 OE cells, whereas the levels were maintained when FOXM1 and MAD2 were overexpressed in MCF7 cells (Supplementary Fig. 4B). Thus, this result suggests that FOXM1 regulates AURORA B allowing MAD2-overexpressing cells to exit mitosis after the induced cell cycle arrest.

To test if FOXM1 overexpression can rescue the detrimental effects of MAD2 OE in vivo, MCF7 cells with empty vector, MAD2 OE or MAD2 together with FOXM1 OE were injected as xenografts in nude mice (Fig. 4D). In line with our in vitro results, persistent high MAD2 levels resulted in decreased fitness and a delayed tumor onset while the combined overexpression of MAD2 and FOXM1 reverted this effect (Fig. 4E). Ten weeks after injection, 2 out of 5 animals injected with MAD2 OE cells developed tumors while the additional overexpression of FOXM1 allowed for 4 out of 5 animals to grow tumors (Fig. 4E). However, FOXM1 did not affect tumor growth at early (day 40) or late (day 56) time points throughout tumor progression (Fig. 4F). Thus, FOXM1 overexpression was not sufficient to accelerate the growth of MAD2 tumors, but it contributed to the tolerance of MAD2-induced detrimental effects on tumor cells.

To corroborate whether FOXM1 endows cells with tolerance to aneuploidy and CIN during chronic MAD2 OE, we analyzed the outcome of cell division in human breast cell lines 20 days after MAD2 induction. MCF7 cells after a long expression of MAD2 continue to present prolonged arrest in mitosis. While long-term MAD2 upregulation resulted in an average mitotic time of 2.67 h, further FOXM1 overexpression was not able to shorten this time (average 2.23 h) (Supplementary Fig. 4C). We next compared CIN levels between MAD2 OE and MAD2/FOXM1 OE by monitoring mitotic errors. A lower percentage of mitotic errors was observed in MAD2 OE cells with constitutive overexpression of FOXM1 indicating a protective role of FOXM1 against CIN during long-term MAD2 OE. (Supplementary Fig. 4D, E). Interestingly, once cells adapted to high MAD2 levels, no cell death was observed in either MAD2 OE cells alone or in the combination with FOXM1 upregulation and the spectrum of mitotic errors was different from the ones seen after a short exposure to MAD2.

### Elevated FOXM1 and aneuploidy are associated with poor prognosis in BRCA patients

**Fig. 5:**
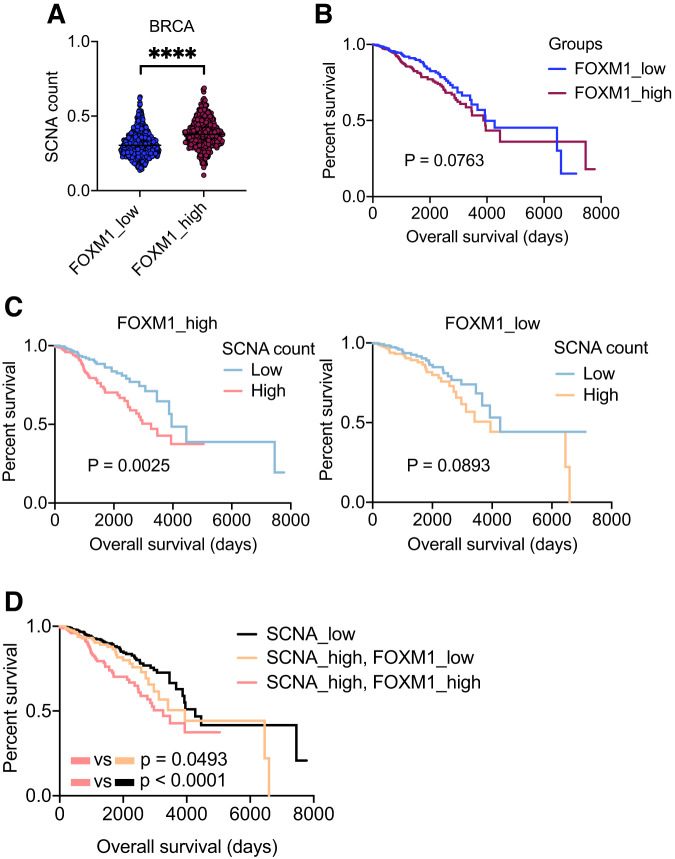
Aneuploid cancer samples expressing high MAD2 have high FOXM1 levels. **A** Mean of copy number changes in FOXM1_low and FOXM1_high BRCA cancer samples from TCGA; *n* = 1064. ****P < 0.0001; Unpaired *t*-test. **B** Survival analysis of FOXM1_low and FOXM1_high BRCA patients. *P* = 0.0763; Long-rank (Mantel-Cox) test. **C** Survival analysis of BRCA patients with low and high SCNA. FOXM1_high cancers (left): *P = 0.0025; FOXM1_low cancers (right): *P* = 0.0893; Long-rank (Mantel-Cox) test. **D** Survival analysis of BRCA patients with low SCNA compared to those with high SCNA and different levels of FOXM1. SCNA_high, FOXM1_high cancers vs SCNA_high, FOXM1_low cancers: *P = 0.0493; SCNA_high, FOXM1_high cancers vs SCNA_low cancers: ****P < 0.0001; Long-rank (Mantel-Cox) test.

Previous data indicated that upregulation of FOXM1 facilitates mitotic exit of MAD2-overexpressing cells in the presence of CIN. We reasoned that aneuploid cancers might also require FOXM1 to compensate for deleterious CIN and hypothesized that cancers that allow propagation of CIN could be associated with a worse survival rate. These results were validated in human breast cell lines and mouse breast tumors, however, to further test this hypothesis, we analyzed TCGA data of BRCA patients (*n* = 1064) and divided them into two groups (FOXM1_low and FOXM1_high) based on *FOXM1* mRNA levels. We then calculated the aneuploidy levels of these samples by determining the mean absolute changes in the copy number segment. Aneuploidy was considered as a deviation from 0, which represents euploidy. FOXM1_low samples showed lower copy number alteration (SCNA) counts while FOXM1_high samples had higher SCNA counts (Fig. 5A). However, no difference in overall survival was observed between these two groups (Fig. 5B). To assess the survival of patients with different aneuploidy levels related to FOXM1 expression, we divided patients into two groups based on SCNA counts (SCNA_low and SCNA_high). Patients with higher SCNA counts presented poor prognoses in FOXM1_high cancers, whereas changes in SCNA had no effect on the prognosis of FOXM1_low cancers (Fig. 5C). Patients with high FOXM1 expression and high SCNA had the worse survival compared to the other groups (Fig. 5D). In summary, these data suggest that survival of BRCA patients is not directly associated with FOXM1 levels. Instead, their outcomes are associated with the upregulation of FOXM1 and increased genomic alterations in the cancers. Thus, FOXM1 might facilitate tolerance of high aneuploid tumors and provide advantages for aneuploid cancer development.

### FOXM1 facilitates mitotic exit after nocodazole-induced arrest

**Fig. 6:**
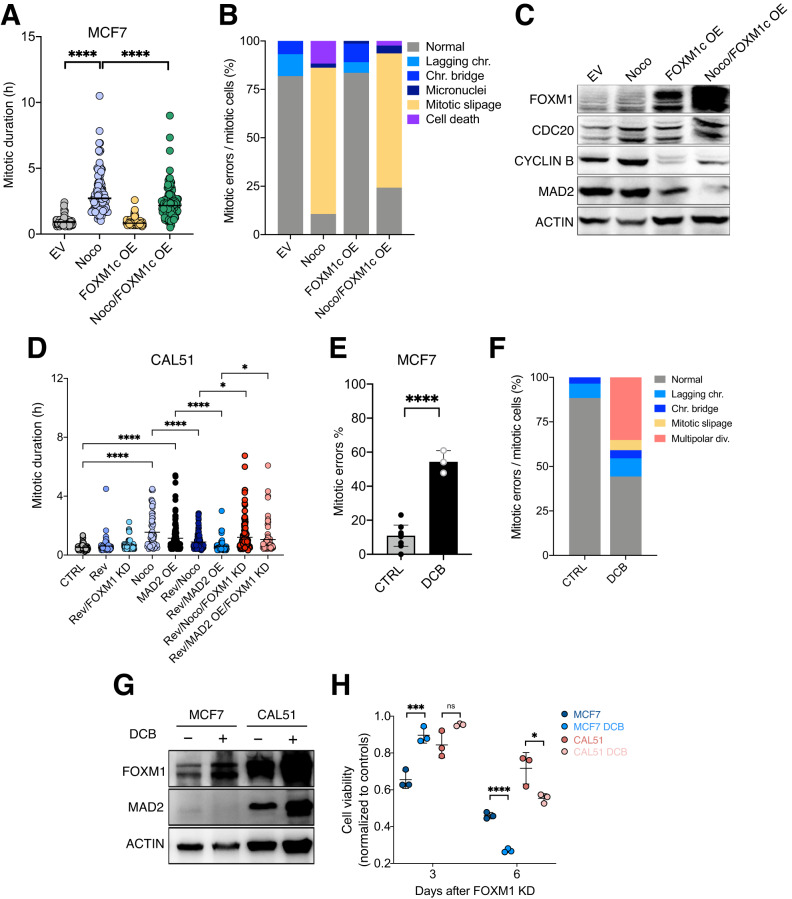
FOXM1 overexpressing cells show increased tolerance after nocodazole-induced mitotic arrest and FOXM1 inhibition impairs fitness of aneuploid cells. **A** Mitotic duration of MCF7 cells after nocodazole (200 μM) treatment, FOMX1 overexpression, and the combination of both for 24 h. ****P < 0.0001; Ordinary one-way ANOVA. (EV, 88 cells; NOCO, 94 cells; FOXM1 OE, 73 cells; NOCO/FOXM1 OE, 124 cells). **B** Cell fate of MCF7 cells with nocodazole (200 μM) treatment or/and FOXM1 overexpression. Data are represented as the incidence of each cell fate/total number of mitotic cells in at least 3 independent movies. (EV, 88 cells; NOCO, 94 cells; FOXM1 OE, 73 cells; NOCO/FOXM1 OE, 124 cells). **C** Western blots showing FOXM1, CDC20, CYLIN B and MAD2 protein levels in MCF7 cells after nocodazole treatment or/and FOXM1 overexpression for 24 h. ACTIN was used as a loading control. Two biological replicates. **D** Mitotic duration of CAL51 cells after Reversine, Nocodazole or MAD2 OE for 3 days and their combination together with si*FOXM1* for 3 days. *P < 0.02, **P = 0.0029, ***P = 0.0002, ****P < 0.0001; Ordinary one-way ANOVA. (CTRL, 69 cells; Rev, 87 cells; Noco, 65 cells; MAD2 OE, 127; Rev/Noco, 139 cells; Rev/MAD2 OE, 78 cells; Rev/Noco/FOXM1 KD, 131 cells; Rev/MAD2 OE/FOXM1 KD, 70 cells). Time lapse microscopy was performed in a minimum of 3 biological replicates. **E** Percentage of mitotic errors in MCF7 cells (CTRL) and after treatment with DCB. ****P < 0.0001 Unpaired *t*-test. At least three biological replicates were analyzed. **F** Cell fate of MCF7 cells (CTRL) or treated with DCB. (CTRL, 112 cells, DCB, 88 cells) three independent movies were analyzed. **G** Representative western blot of FOXM1, MAD2 and ACTIN in human cell lines after treatment with dihydrocytochalasin B (DCB). 3 biological replicates. **H** Cell viability of MCF7 and CAL51 with or without DCB treatment and FOXM1 inhibition with siRNA for 3 or 6 days. Cell viability of each cell line was normalized to that of each untreated group (*n* = 3 independent experiments). ****P < 0001, ***P = 0.0028, *P = 0.0335; Unpaired *t*-test. For original and additional wbs see Supplemental Material. Raw data for A,B,D,E,F is included in Supplemental Tables.

We speculated that increased FOXM1 levels antagonize mitotic checkpoint signaling and release cells from mitotic arrest. To test this hypothesis, we forced mitotic checkpoint activation by treating cells with the spindle poison nocodazole (Noco). A high dose of Noco treatment blocked cells in mitosis and in line with our previous results, FOXM1 overexpression released these cells from prolonged mitosis (Fig. 6A). In addition, high levels of FOXM1 partially rescued the mitotic slippage induced after Noco treatment (75% of mitotic slippage in Noco treated cells vs 68% in Noco/FOXM1overexpression), as well as cell death in mitosis (11% vs 2%) (Fig. 6B). SAC effectors including MAD2 and CDC20 are all involved in chromosome segregation [37]. Cyclin B is known to block mitotic exit regardless of SAC activity and is degraded by the CDC20-APC/C complex during mitosis [35]. We confirmed MAD2 and Cyclin B protein levels to be downregulated in Noco-treated FOXM1 OE cells compared to control cells after Noco treatment (Fig. 6C). This suggests that FOXM1 OE compromises SAC signaling and reduces Cyclin B in MCF7 cells, leading to chromosome segregation and exit from mitosis.

Next, to test whether FOXM1 is responsible for allowing aneuploid cells to exit mitosis, we challenged CAL51 cells with Reversine, an MPS1 inhibitor that abrogates SAC signaling and promotes premature exit from mitosis [38]. While Reversine treatment in control cells or FOXM1 KD cells did not alter mitotic duration, mitotic arrest induced by MAD2 or Noco- was reverted after Reversine treatment in CAL51 cells (Fig. 6D). Importantly, Reversine was not able to rescue the prolonged mitosis in CAL51 cells when si*FOXM1* was applied (Fig. 6D). These data suggest that FOXM1 is crucial to restoring mitotic exit in aneuploid cells with a hyperactivated SAC.

To further verify if FOXM1 is essential for aneuploid cells, we attempted to generate aneuploid cells by treating breast cancer cells with dihydrocytochalasin B (DCB), to block cytokinesis and induce tetraploidy [39]. Consistent with the previous reports [40, 41], an increase of mitotic errors was observed after DCB treatment, suggesting that cells became chromosomally unstable (Fig. 6E, F). MCF7 and CAL51 cell lines exhibited increased FOXM1 protein levels after DCB treatment. In addition, CAL51 cells treated with DCB also had higher MAD2 levels compared to non-treated cells (Fig. 6G). We next inhibited FOXM1 in these unstable cells. Long-term inhibition of FOXM1 impaired proliferation, especially in the DCB-treated cells (Fig. 6H) suggesting that chromosomally unstable cells have increased sensitivity to FOXM1 inhibition.

## Discussion

In our current study, we report that depletion of *Foxm1* in various cells that overexpress MAD2 (including human breast tumor cells, MEFs, normal mouse mammary epithelial cells and mouse tumor cells), leads to an extension of mitosis and an increase in mitotic errors. Tumor cells with high levels of MAD2 can adapt over time by accumulating FOXM1, which renders them vulnerable to FOXM1 inhibition. Our investigation has revealed that FOXM1 upregulation facilitates chromosome segregation and mitotic exit in human cell lines with high MAD2 levels by suppressing SAC signaling. As a result, these cells are rescued from their defective proliferation. Additionally, we have found that FOXM1 can reverse mitotic arrest and correct mitotic errors induced by other mechanisms such as nocodazole or cytokinesis failure.

Tumors can overcome the mitotic checkpoint induced by Mad2 overexpression and become aneuploid [12, 13]. The increasing number of mitotic errors suggest that cells force mitotic exit and regain cell fitness despite acquiring CIN. Interestingly, high levels of CIN can actually be tumor-suppressive, making high CIN tumors a potential therapeutic opportunity to halt CIN accumulation in unstable cells [5].

MAD2 overexpression in cells is detrimental, but the mechanisms by which cells overcome this effect are not well understood. By analyzing the genetic dependencies in high MAD2 aneuploid cell lines we found that FOXM1 is an essential gene in these cells. FOXM1 can activate various cell cycle-related proteins and is closely associated with CIN, suggesting that FOXM1 might be relevant to tolerate chromosomal instability. Our findings support the idea that high MAD2 CIN cells might require FOXM1 for maintaining proliferative fitness. However, once MAD2 is overexpressed for an extended period of time, chromosome errors persist, and the number of polyploid cells increases, suggesting that cells can escape the mitotic arrest via slippage. Although multiple mechanisms can allow cells to escape from mitotic arrest [15, 42], we found that the level of SAC proteins decreases in MAD2 overexpressing cells with FOXM1 upregulation, providing insights into the role of FOXM1 in the maintenance of chromosomal stability.

Similarly, inhibition of FOXM1 increases the levels of these SAC proteins, strengthening the checkpoint and inducing mitotic arrest and cell death. We conclude that FOXM1 is required for proliferative fitness in high MAD2 cells with CIN. Nevertheless, FOXM1 prevents mitotic cell death in cells with paclitaxel treatment [21], our results cannot exclude the possibility of cell death prevention of FOXM1during MAD2 overexpression.

FOXM1 insufficiency causes centrosome abnormalities and disrupts the spindle formation [43, 44]. Thus, FOXM1 is a valuable target to increase mitotic stress in CIN cells. Consistent with this, we find that short-term FOXM1 inhibition is insufficient to arrest cells in mitosis but leads to lagging chromosomes and chromosome misalignments in human cell lines. As mitotic errors accumulate, several cellular outcomes are expected. First, the mitotic checkpoint is activated leading to mitotic arrest [11]. Second, DNA damage and replication stress block cell cycle entry, leading to quiescence and senescence [45]. Finally, overwhelmingly high CIN levels activate apoptotic pathways and induce cell death [46]. In agreement, our results demonstrate that high levels of MAD2 confer aneuploid cell lines sensitivity to FOXM1 inhibition, reducing their proliferative ability as long-term FOXM1 inhibition induces high levels of mitotic aberrations. Thus, FOXM1 represents a critical target to tumor cells exhibiting CIN or aneuploidy.

## Supplementary information

Supplementary fugures and legends

Supplementary Table CAL51 movies

Supplementary Table Figure 2I and J movies

Supplementary Table MCF7 movies

Original Data File

checklist

## Data Availability

All data generated and analyzed during the study are available from the corresponding author upon reasonable request.
