# Supplementary material for: FOXM1 is critical for the fitness recovery of chromosomally unstable cells
Source: Cell Death Dis. 2023 Jul 14;14(7):430. doi: 10.1038/s41419-023-05946-2 (PMC10349069; doi:10.1038/s41419-023-05946-2)
Supplement: Supplementary file 1 — Supplementary fugures and legends [file 41419_2023_5946_MOESM1_ESM.pdf]

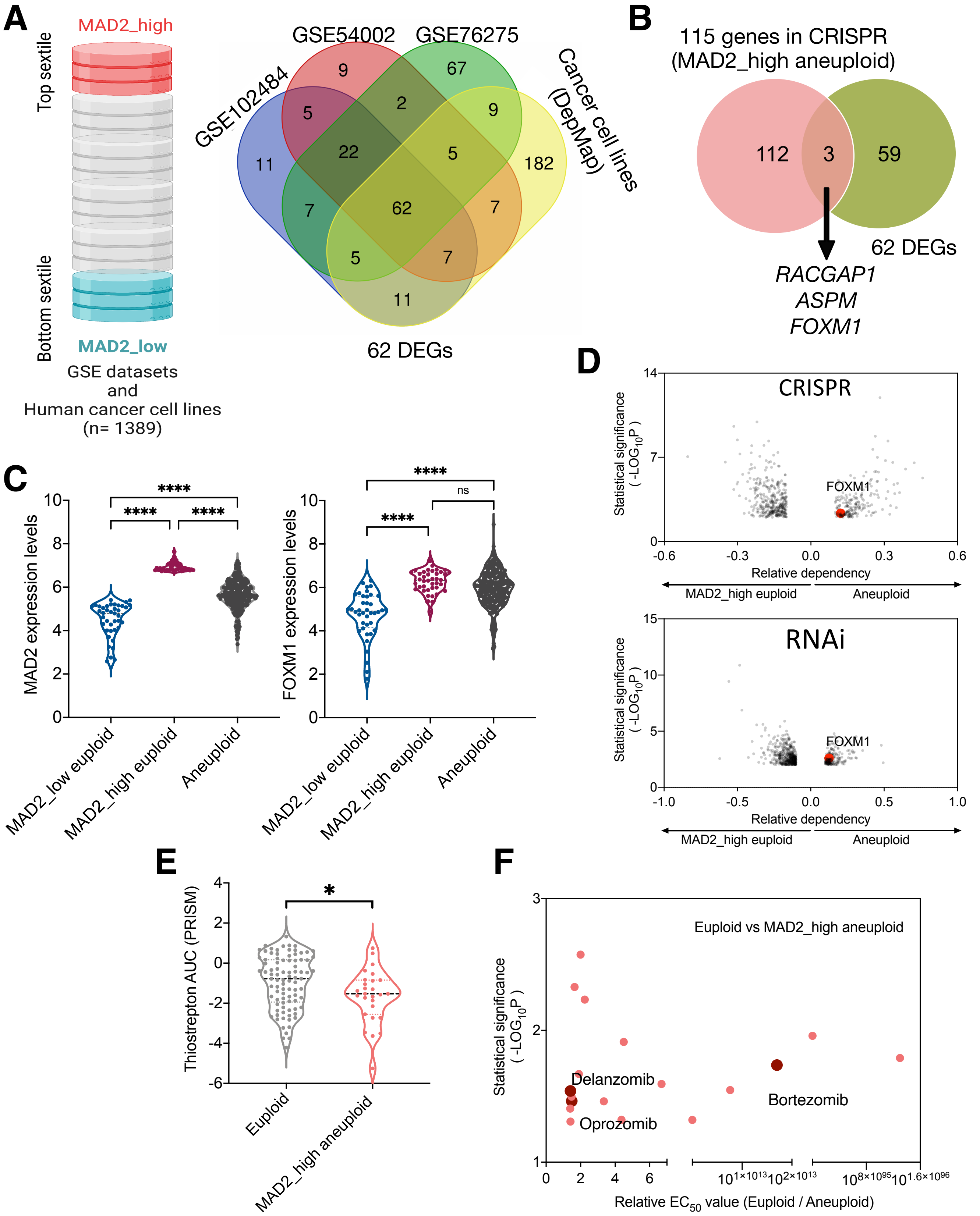

Supp Figure 1

Supplementary Figure 1. High levels of aneuploidy sensitize cancer cells to FOXM1 inhibition

A) On the left, schematic of the distribution of GSE tumors and cancer cell lines (DepMap) into MAD2_high and MAD2_low groups. On the right, Venn diagram showing the upregulated genes in the MAD2_high GSE tumors and cell lines after comparison with the corresponding MAD2_low group. The genes that were common in all comparisons were considered as differentially expressed genes (DEGs). **B**) Venn diagram showing the overlap between the DEGs and the essential genes for MAD2_high aneuploid from a CRISPR-Cas9 screening. **C**) *MAD2* and *FOXM1* mRNA expression levels in MAD2_high or low euploid and aneuploid cancer cell lines. ****P<0.0001; One-way ANOVA. **D**) Essential genes in MAD2_high euploid and aneuploid cell lines in a CRISPR-Cas9 screen and RNAi datasets shown as relative dependency. FOXM1 is highlighted in red. **E**) The area under the fitted dose response curve (AUC) represents drug sensitivity of euploid and aneuploid cancer cell lines (MAD2_high and low) to the FOXM1 inhibitor Thiostrepton (PRISM repurposing primary screen), *P=0.0272; One-way ANOVA. **F**) Half maximal effective concentration (EC50) of MAD2_high aneuploid cancer cell lines in drug sensitivity dose-level (PRISM repurposing secondary screen).

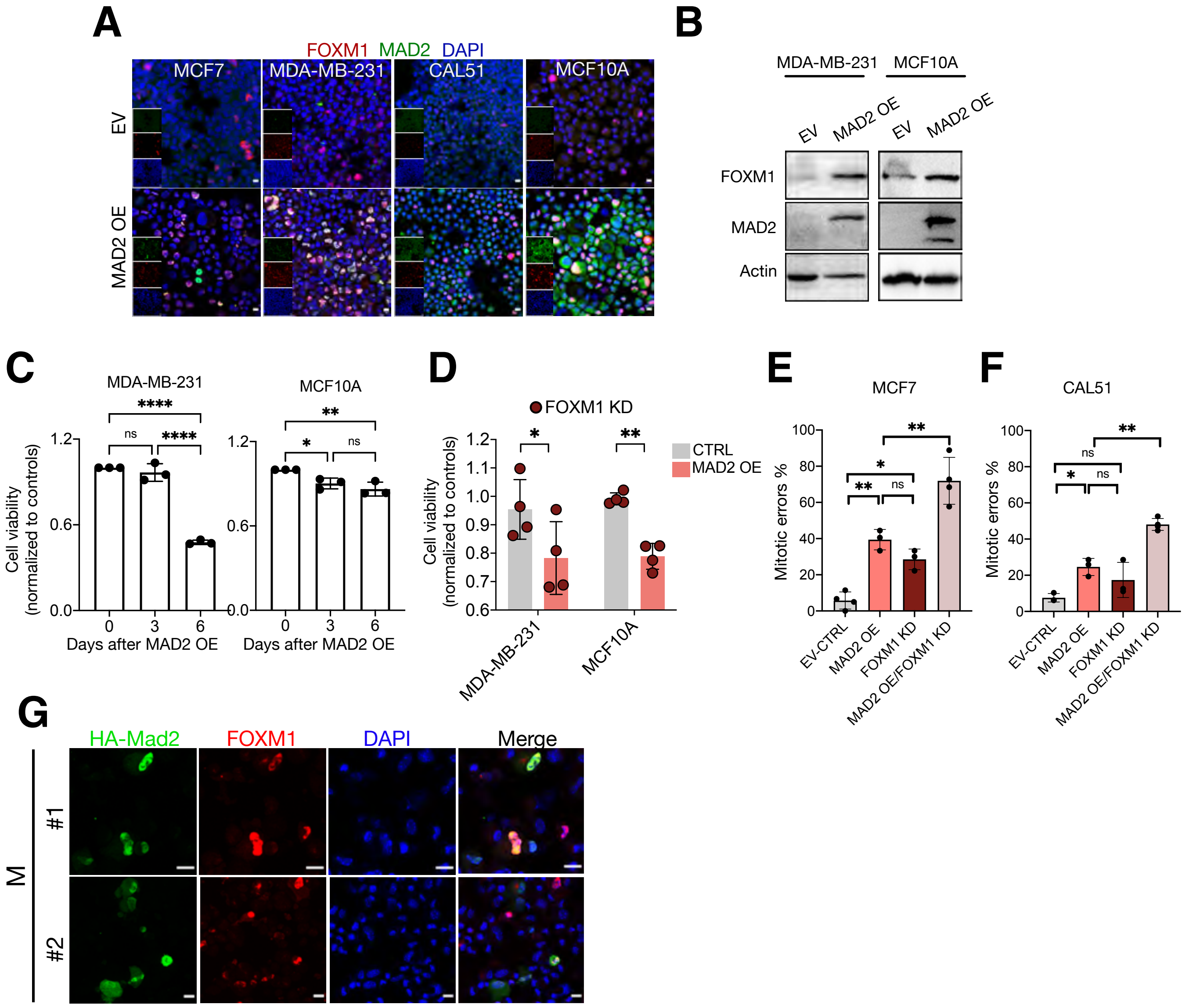

Supp Figure 2

Supplementary Figure 2. Expression and cell viability of human breast cancer cell lines and 3D cultures with FOXM1 inhibition

A) Immunofluorescence of FOXM1 and MAD2 on control (EV) or MAD2 overexpressing human cell lines after 6 days on dox. Scale bar: 10μm. **B**) Western blots of FOXM1 and MAD2 in MDA-MB-231 and MCF10A cell lines infected with an empty vector (EV) or a Dox-inducible MAD2 expressing vector (MAD2 OE) after dox administration for 6 days. ACTIN was used as a loading control. 3 biological replicates. **C**) Cell viability of human cell lines after dox treatment for 3 or 6 days. Each dox-treated cell line was normalized to the untreated one. MDA-MB-231: ***P<0.0001, MCF10A: **P<0.008, *P=0.0359, One-way ANOVA. Each dot is a biological replicate. **D**) Cell viability of human cell lines after MAD2 overexpression and *FOXM1* knockdown by siRNA for 6 days. Values of each cell line were normalized to those of each EV group (n=4). MDA-MB-231: *P=0.0189; MCF10A: **P=0.0047; Two-way ANOVA. **E**) Percentage of mitotic errors in MCF7 cells after MAD2 OE, FOXM1 KD and both. *P=0.02, **P=0.0017. One-way ANOVA. **F**) Percentage of mitotic errors in CAL51 cells after MAD2 OE, FOXM1 KD and both. *P=0.019, **P=0.0017, One-way ANOVA. **G**) Immunofluorescence staining of EP cells after Mad2 induction. Cells from two different mice were used. Scale bar: 10 μm.

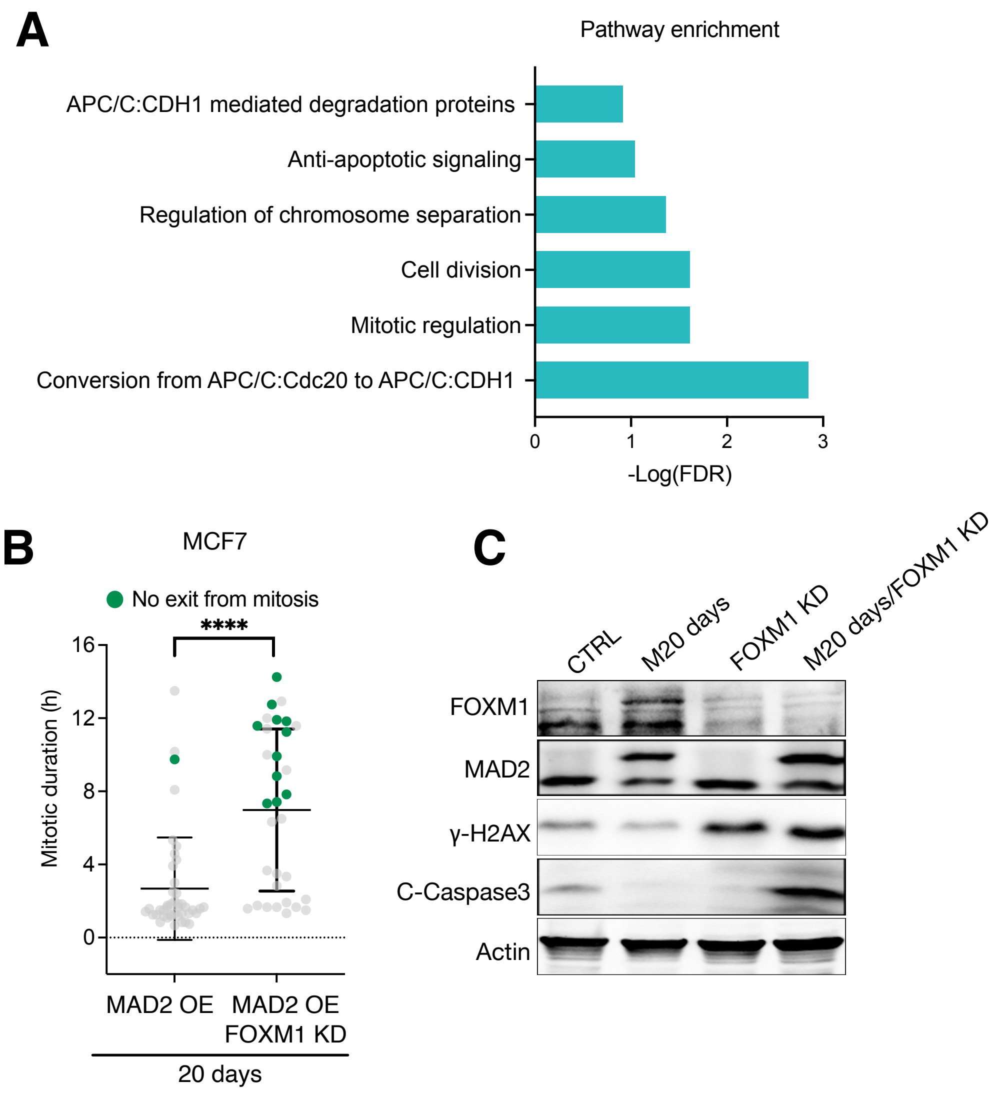

Supp Figure 3

Supplementary Figure 3. Consequences of long term MAD2 overexpression

A) Metascape analysis of pathways enriched in K tumors when compared to KM tumors. **B**) Mitotic duration of MCF7 cells with long-term MAD2 overexpression (20 days) and after si*FOXM1* for 3 days. (MAD2;43 cells, MAD2/FOXM1 KD; 32 cells. ****P<0.001; Unpaired t-test. Green dots represent cells that did not complete mitosis. **C**) Western blots of FOXM1, MAD2, gamma-H2AX, and Cleaved-caspase3 in long-term MAD2 overexpressing MCF7 cells after treatment with siRNA against *FOXM1* for 3 days. Actin was used as a loading control.

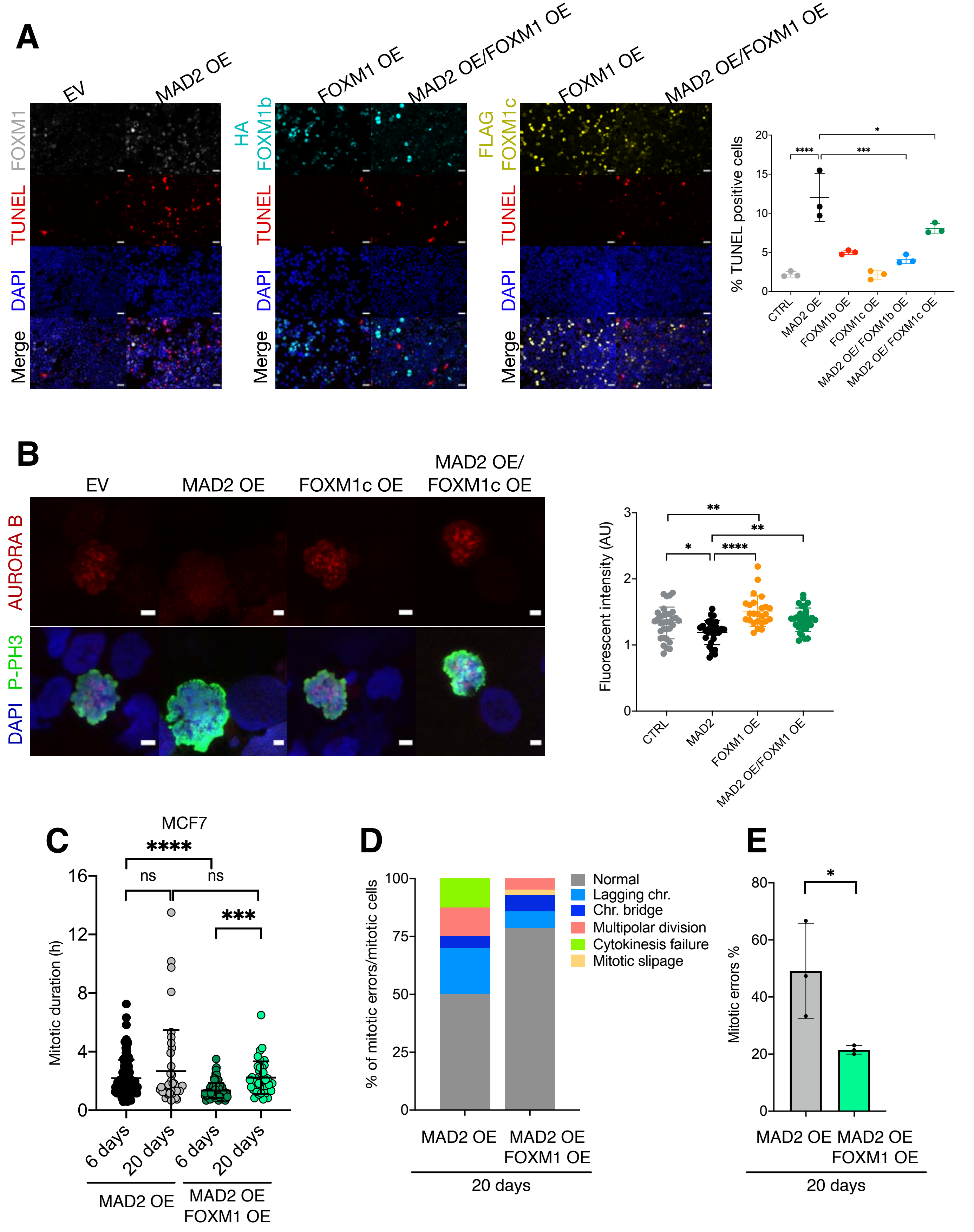

Supp Figure 4

Supplementary Figure 4. FOXM1 is required for timely mitosis in long-term MAD2 tolerance

A) Representative images of TUNEL staining. FOXM1, HA-tagged FOXM1 and FLAG-tagged FOXM1 on each cell line after 6 days on Dox. Scale bars: 10 μm. Quantification of TUNEL-positive MCF7 cells after FOXM1 and MAD2 overexpression for 6 days (n=3). ****P<00001, ***P=0.0001, *P=0.0306; Ordinary one-way ANOVA. **B**) Representative images of p-PH3 and quantification of AURORA B immunofluorescent staining in MCF7 cells. Scale bar: 5 μm. Cell number: EV, 33 cells; MAD2 OE, 31 cells; FOXM1b OE, 25 cells; MAD2 OE/FOXM1b OE, 31 cells. ****P<0001, **P<0.01, *P=0.0296; Ordinary one-way ANOVA. **C)** Mitotic duration of MCF7 cells with short (6 days) and long-term MAD2 overexpression (20 days) and after FOXM1 OE (MAD2, 6 days 220 cells, MAD2 20 days, 43 cells, MAD2/FOXM1 OE 6 days, 130 cells; MAD2/FOXM1 OE 20 days, 42 cells. **P=0.0013, ****P<0.001; One-way ANOVA. Data from 3 independent movies. **D**) Cell fate shown as the percentage of mitotic errors in MCF7 cells with long-term MAD2 overexpression or MAD2 and FOXM1 overexpression. Same cells as in (C). **E)** Percentage of mitotic errors after long-term MAD2 overexpression (20 days) and after FOXM1 OE. Same cells as in (C). *P=0.046, Unpaired t test.
