# Supplementary material for: FOXM1 is critical for the fitness recovery of chromosomally unstable cells
Source: Cell Death Dis. 2023 Jul 14;14(7):430. doi: 10.1038/s41419-023-05946-2 (PMC10349069; doi:10.1038/s41419-023-05946-2)

Figure 2A: Western blots of FOXM1 and MAD2 in MCF7 and Cal51cell lines infected with an empty vector (EV) or a Dox-inducible MAD2 expressing vector (MAD2 OE) after dox administration for 6 days . The dotted blot is shown in figure 2A. The blue squares represent additional samples.

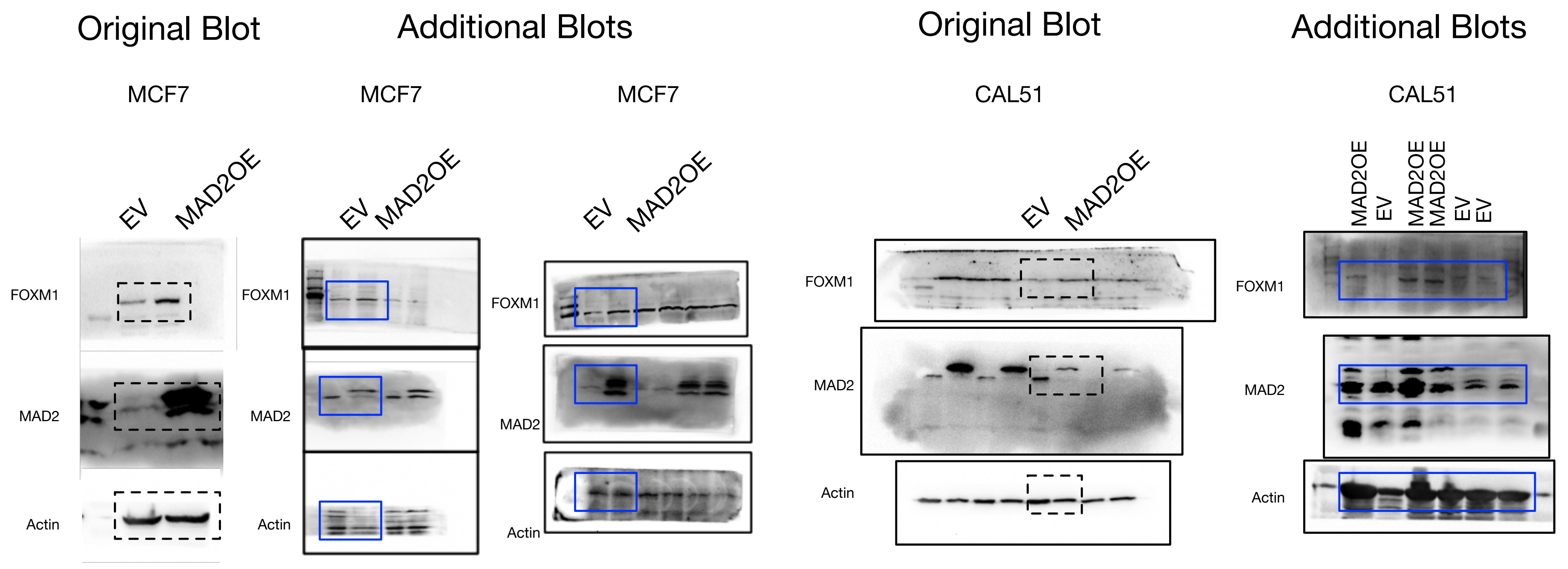

Supplementary Figure 2A: Western blots of FOXM1 and MAD2 in MDA-MB-231 and MCF10A lines infected with an empty vector (EV) or a Dox-inducible MAD2 expressing vector (MAD2 OE) after dox administration for 6 days .The dotted blot is shown in Supplementary figure 2A. The blue squares represent additional samples.

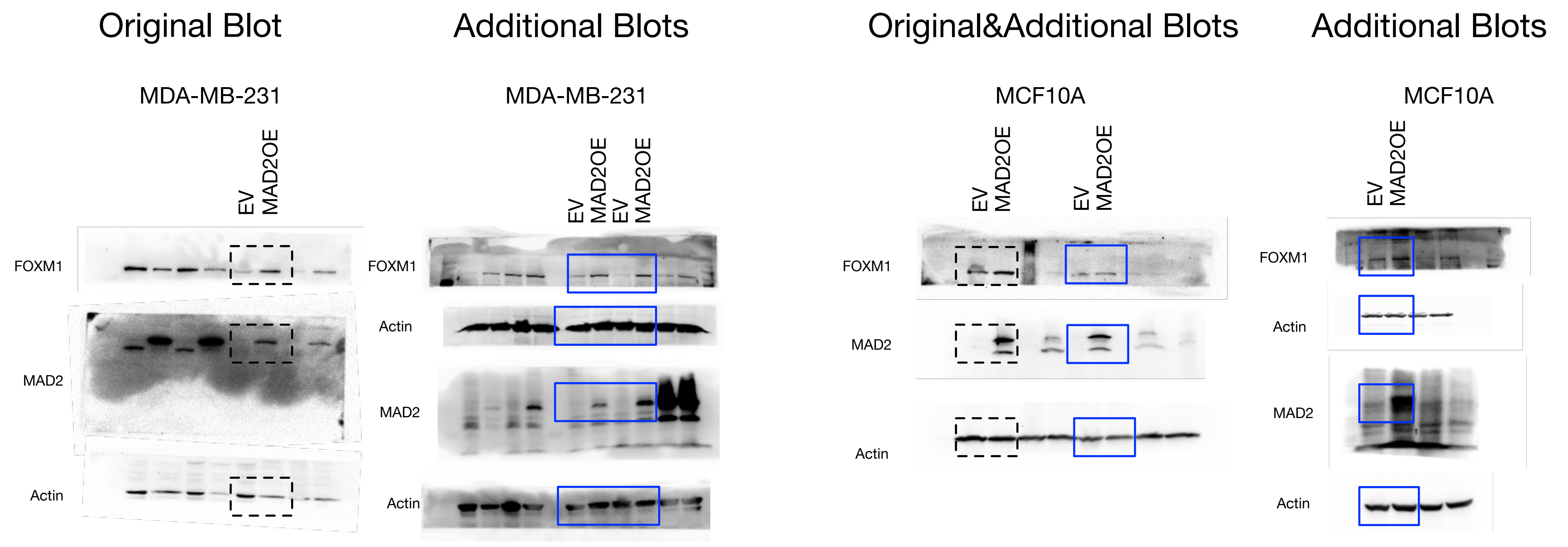

Figure 2H: western blot of *KH2-HA-Mad2/Rosa26-rtTA* MEFs (5 MEFs lines) that were either not induced (CTRL) or on Dox for 30 hours. The dotted blot is shown in figure 2H. The blue squares represent additional samples used for the quantification.

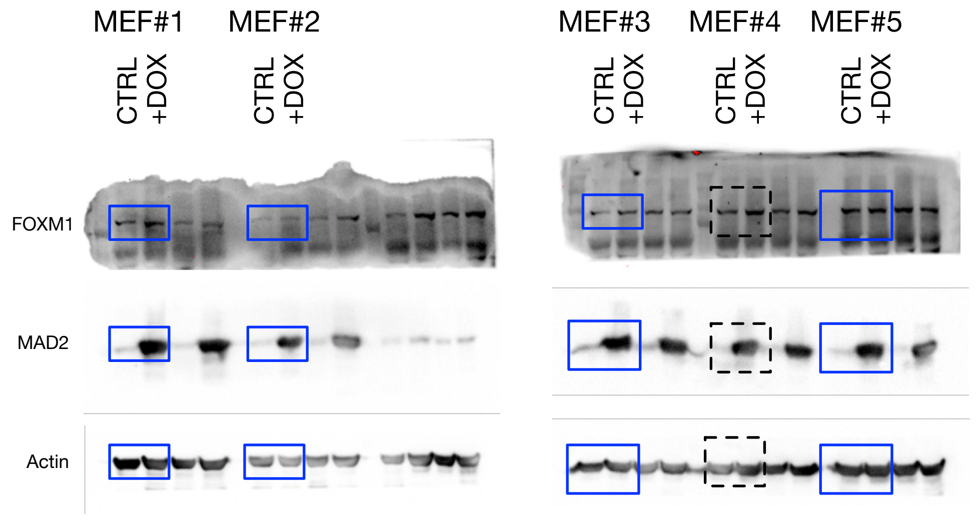

Figure 2K: western blot of FOXM1 and HA-Mad2 in EP cells from two *TetO-Mad2/MMTV-rtTA* Dox-inducible transgenic mice (M1 and M2) without DOX (CTRL) and with DOX

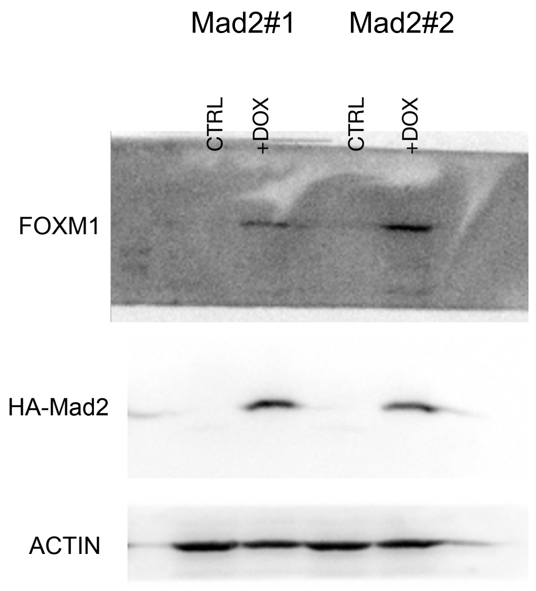

Figure 3A: Western blot of FOXM1 and HA-Mad2 in Kras (K1, K2, K3) and Kras/Mad2 (KM1, KM2, KM3) breast tumor cells. Dotted black lines show the western blots in Figure 3A. The additional blots were done (n=9 K and 9 KM tumors) for the quantification but not shown in the figure. Blue lines

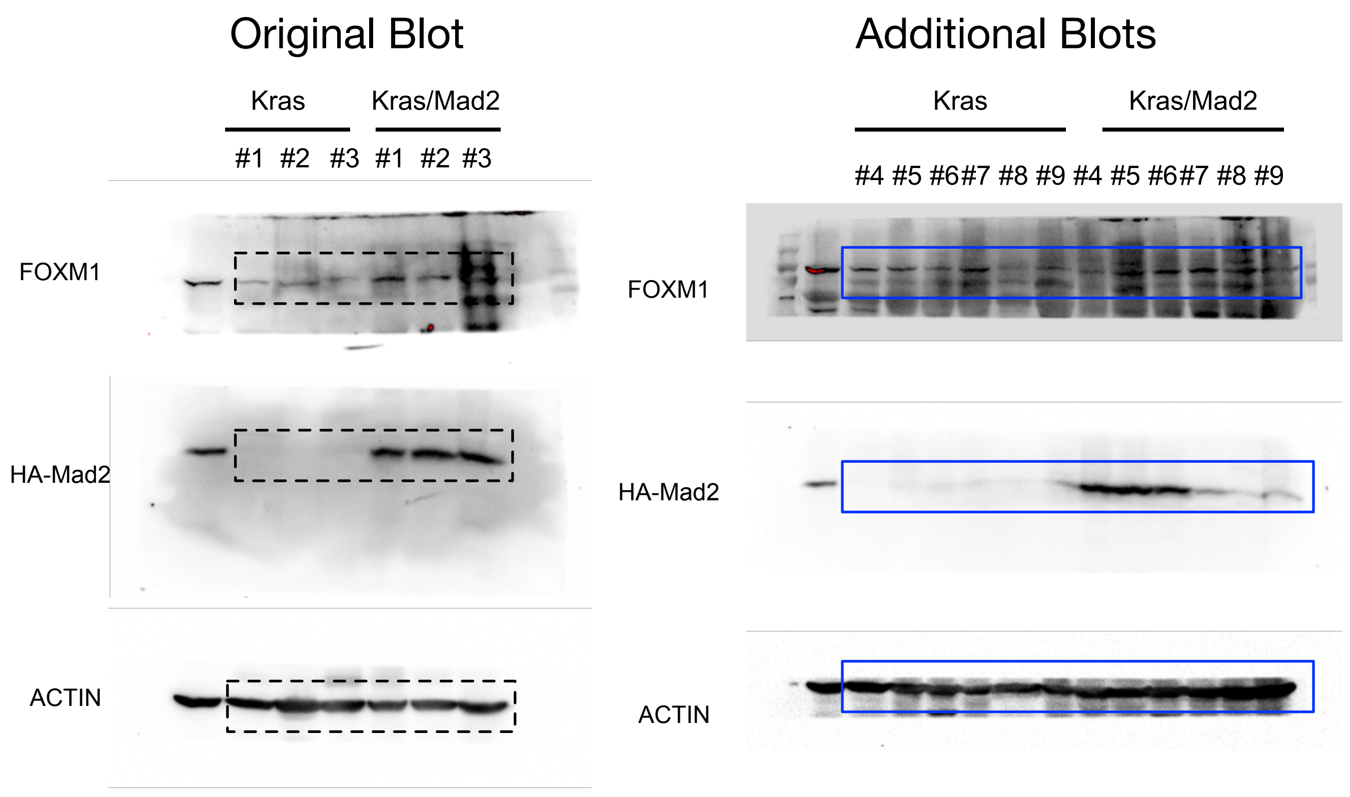

Figure 3F: Western blots of FOXM1, HA-Mad2, Cleaved-caspase3 (C-Casp3) and gama-H2AX in K and KM tumor cells after RCM-1 treatment or si*Foxm1* for 6 days. ACTIN was used as a loading control. Dotted black lines show the western blots in Figure 3F. Blue lines are additional blots.

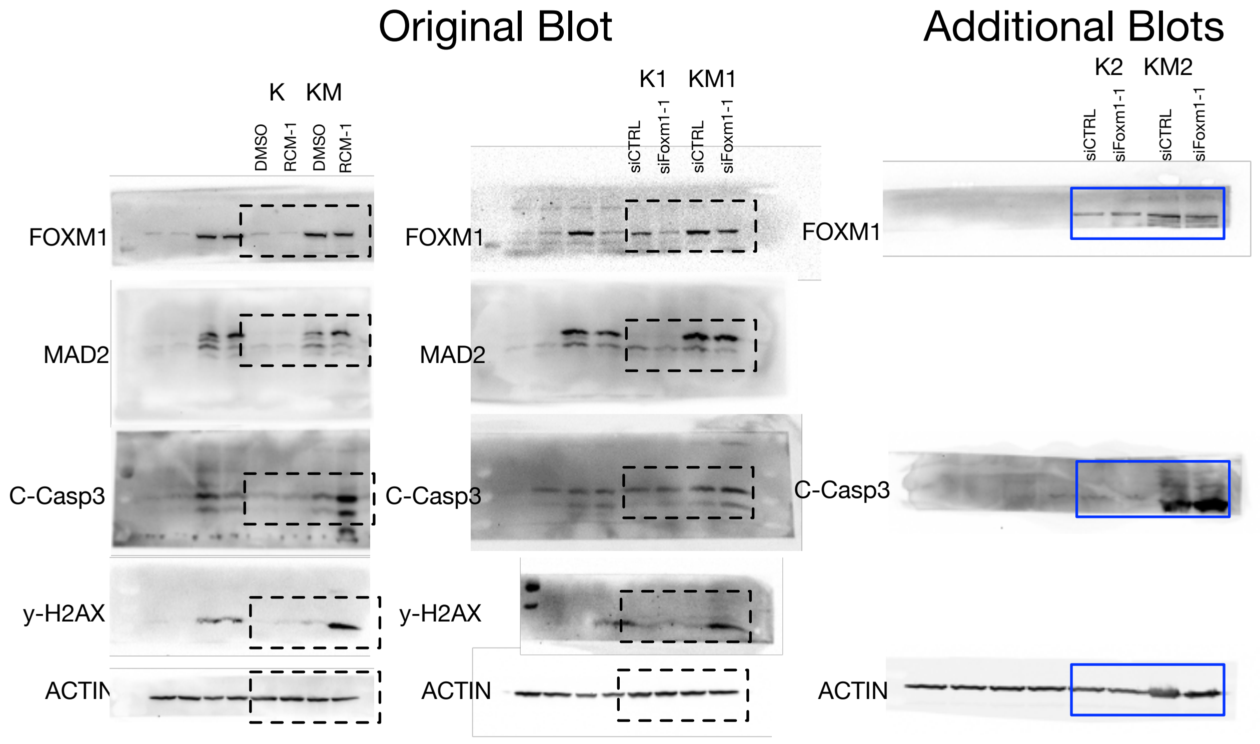

Supplementary Figure 3C; Western blots of FOXM1, MAD2, gamma-H2AX, and Cleaved-caspase3 in long-term MAD2 overexpressing MCF7 cells after treatment with siRNA against *FOXM1* for 3 days. Actin was used as a loading control. Dotted black lines show the western blots in Supplementary Figure 3C.

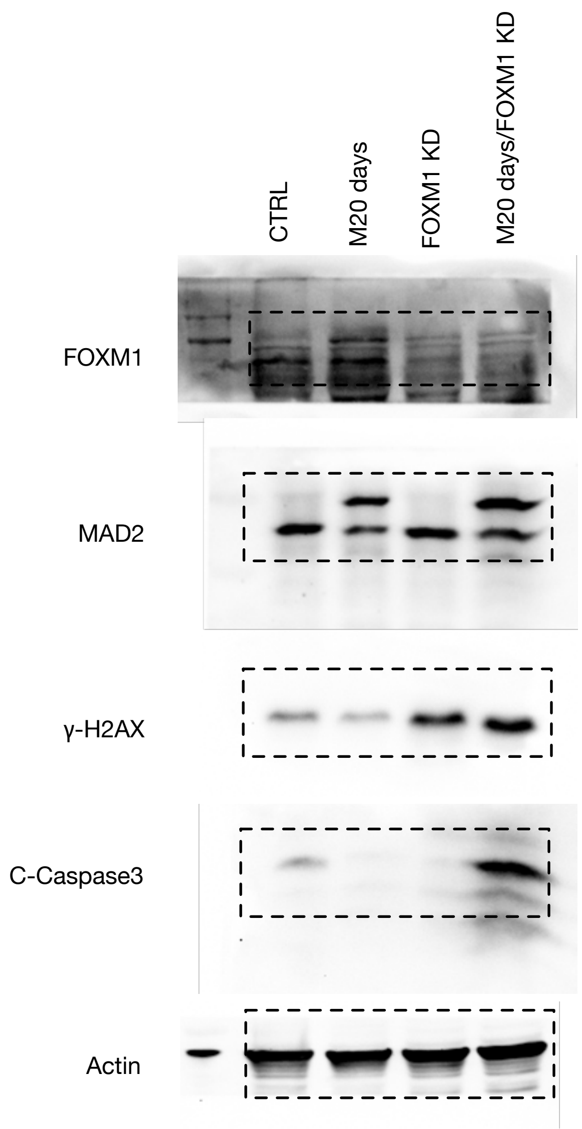

Figure 6C: Western blots showing FOXM1, CDC20, CYLIN B and MAD2 protein levels in MCF7 cells after nocodazole treatment or/and FOXM1 overexpression for 24 hours. ACTIN was used as a loading control. Dotted black lines represent the original blot shown in Figure 6C. Blue lines show additional blots.

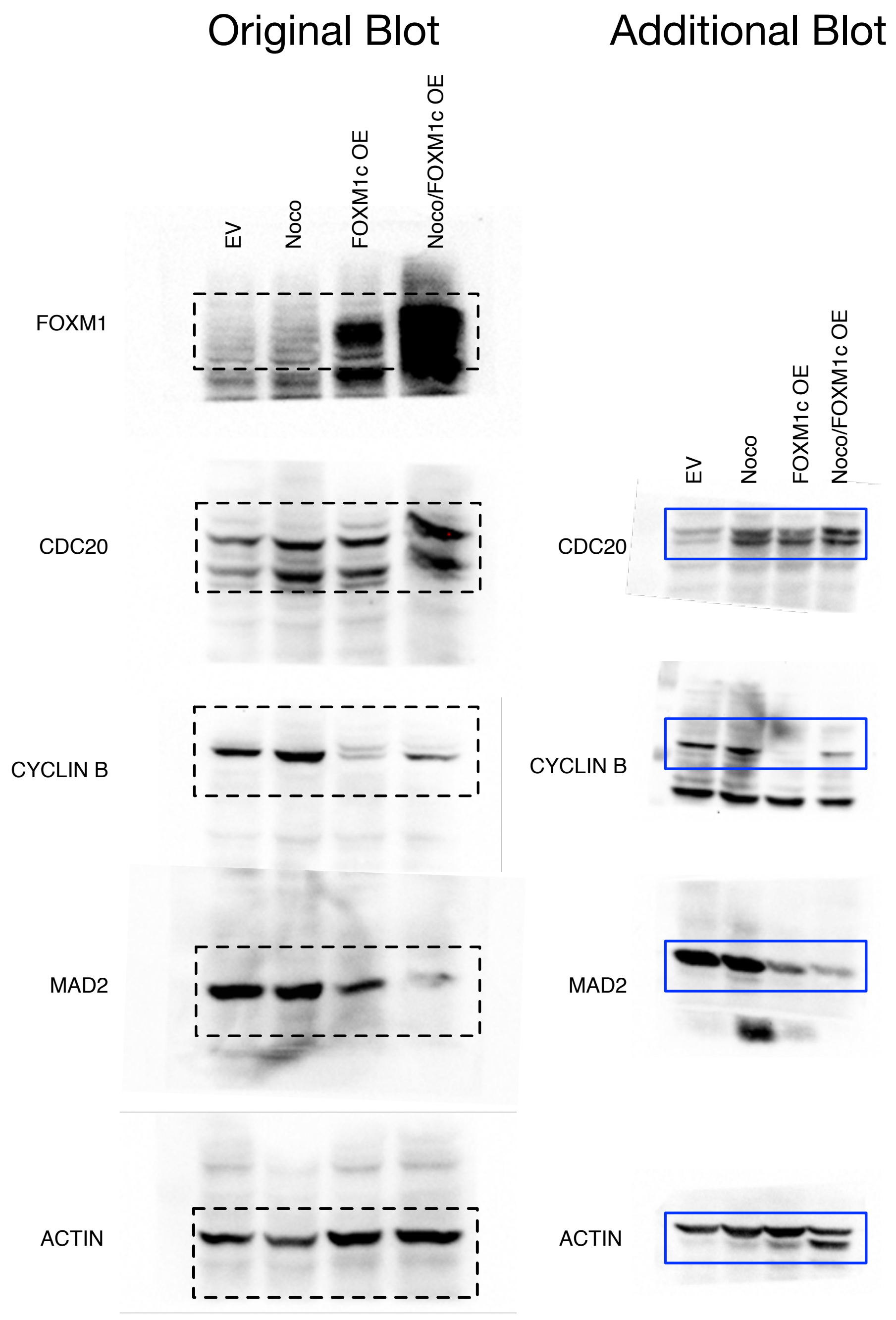

Figure 6F: Representative western blot of FOXM1, MAD2 and ACTIN in human cell lines after treatment with dihydrocytochalasin B (DCB). Dotted black lines show the western blots in Figure 6F. Blue lines are additional blots.

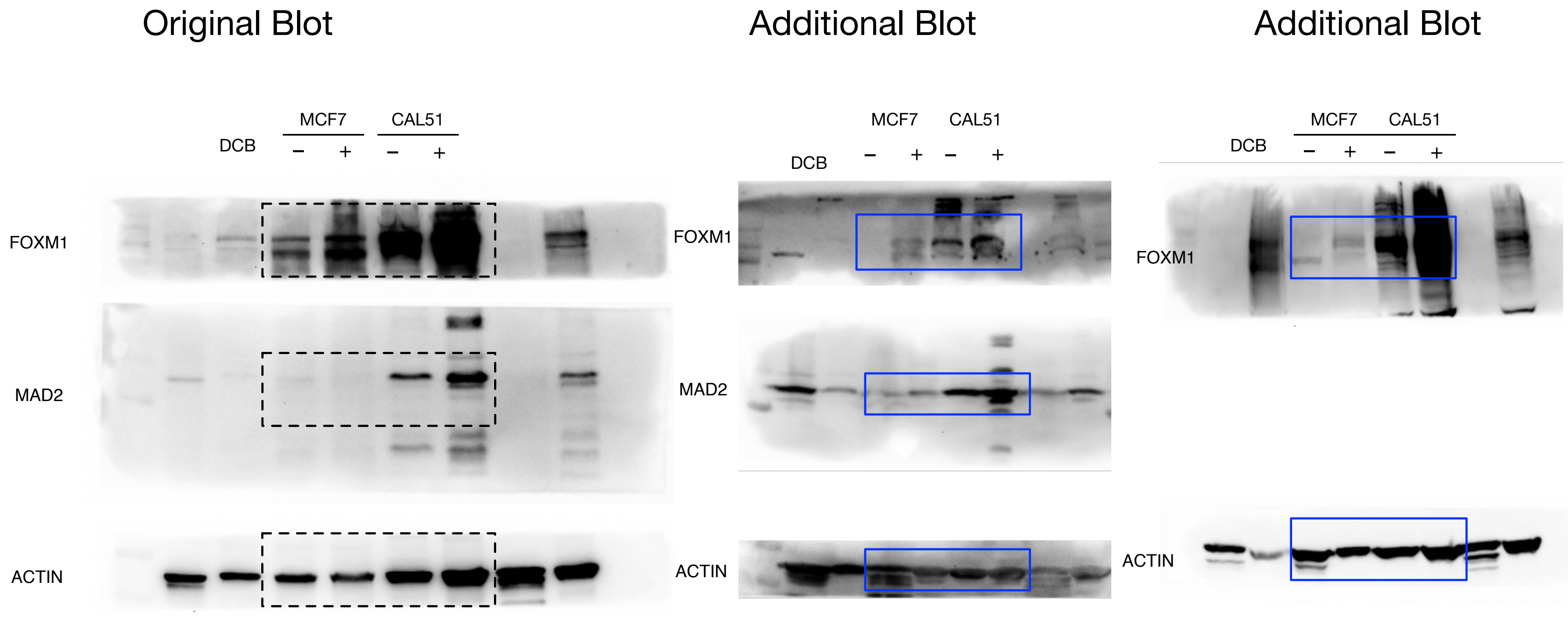

Supplement: Supplementary file 5 — Original Data File [file 41419_2023_5946_MOESM5_ESM.pdf]
